# Insights into Neanderthal development, childbirth, and locomotion from the Palomas and Dederiyeh pelves

**DOI:** 10.1073/pnas.2535303123

**Published:** 2026-08-17

**Authors:** Christoph P. E. Zollikofer, Marcia S. Ponce de León, María Haber Uriarte, Mariano V. López, Jon Ortega, Osamu Kondo, Takeru Akazawa, Michael J. Walker

**Affiliations:** ^a^ Department of Informatics, University of Zurich, Zurich 8050, Switzerland; ^b^ Center for Climate Physics, Institute for Basic Science, Busan 46241, Republic of Korea; ^c^ Department of Prehistory, Archaeology, Ancient History, Mediaeval History and Historiographical Studies, Faculty of Letters, University of Murcia, Murcia 30001, Spain; ^d^Murcian Association for the Study of Palaeoanthropology and the Quaternary, Murcia 30008, Spain; ^e^ Department of Biological Sciences, Graduate School of Science, University of Tokyo, Tokyo 113-0033, Japan; ^f^ Research Institute, Kochi University of Technology, Kochi 782-8502, Japan

**Keywords:** Neanderthal pelvis, obstetrical dilemma, bipedal locomotion, virtual reconstruction, sexual dimorphism

## Abstract

The human pelvis reflects evolutionary compromises among childbirth, bipedal locomotion, and other factors such as pelvic floor stability and thermoregulation. However, limited fossil evidence has hindered understanding of how these factors shaped pelvic morphology in Neanderthals, our closest extinct relatives. Here, we present new virtual reconstructions and morphometric analyses of nearly complete Neanderthal pelves from Sima de las Palomas del Cabezo Gordo (Spain) and Dederiyeh Cave (Syria), comparing them with other Neanderthals and modern humans. We find that Neanderthal and human pelves differ in overall shape from an early age but share similar sexual dimorphism patterns with humans. Pelvic regions associated with childbirth exhibit comparable functional morphology, suggesting similar birth mechanisms. Conversely, locomotor-related features such as hip joint orientation and muscle moment arm geometry differ, indicating distinct stance and gait adaptations. Our findings are consistent with the hypothesis that evolutionary constraints traditionally framed as an “obstetrical dilemma” may have involved a trade-off between childbirth and pelvic floor stability, rather than between childbirth and locomotor performance.

## Significance

For decades, the “obstetrical dilemma” has been used to explain the shape of the human pelvis as a compromise between giving birth to large-brained babies and walking efficiently on two legs. Exceptionally preserved Neanderthal pelves allow us to test this idea in deep time. Our analyses show that pelvic regions important for childbirth are remarkably similar in Neanderthal and human females, while those linked to locomotion differ. This pattern suggests that, for at least the past several hundred thousand years, the main evolutionary constraint on pelvic form may have been between childbirth and maintaining pelvic floor stability, rather than between childbirth and locomotion—offering a revised perspective on a long-standing evolutionary hypothesis.

The modern human pelvis is distinguished by a suite of derived morphological features reflecting adaptation to bipedal locomotion and stance and to the demands of giving birth to large-brained, large-bodied babies. Its pronounced sexual dimorphism has been interpreted traditionally through the lens of the Obstetrical Dilemma Hypothesis (ODH) (1), which posits a functional conflict between the need for a wide birth canal and the need for a narrow distance between the femoral head centers (FHC distance) required for efficient bipedal locomotion. The ODH has received both criticism and support from diverse perspectives. Its validity has been questioned in the light of evidence emphasizing maternal metabolic constraints rather than pelvic morphology as limiting factors (2), as well as data suggesting that locomotor efficiency does not correlate with FHC distance (3–6). However, recent studies have provided renewed support by drawing on pelvic floor stability models (7) and theoretical models such as the “cliff-edge hypothesis,” which addresses evolutionary mismatches between maternal pelvic dimensions and neonatal cranial size (8). Integration of these aspects into a genome-wide association study (GWAS) of the human pelvic phenotype and associated health data (9) has provided support for the “multifactor pelvis hypothesis” (MPH) (10), which posits that pelvic form is optimized under the often conflicting demands of childbearing/childbirth, locomotor performance, pelvic floor stability, and thermoregulation.

The ODH and MPH address aspects of an even broader multifactorial optimization process—one that likely predates the evolution of obligate bipedalism (11, 12). On the one hand, human-like pelvic sexual dimorphism (PSD) is evident across extant great ape species (13, 14), albeit to a lesser degree than in humans, on the other, trade-offs involving maternal-to-neonate body size, obstetrics, locomotor biomechanics, and metabolism appear widespread among primates (15–18).

Although the pelves of early hominins exhibit morphological adaptations for bipedalism (19), identifying their obstetrical adaptations remains challenging (20–23). Fossil evidence from *Australopithecus* and early *Homo* suggests a transverse ellipsoid shape of their pelves (wide transverse relative to anteroposterior dimensions) compared to modern humans, giving rise to the hypothesis that their relatively large-brained neonates were delivered via a transverse passage through the entire birth canal (21, 22, 24). Within this framework, the rotational birth mechanism typical of modern humans—where the neonate executes a quarter-turn during passage from inlet to outlet—traditionally has been thought to have emerged later in hominin evolution (24, 25), perhaps in parallel with the evolution of a transversely narrow pelvis specialized for endurance running (26) [but see (27, 28) for alternative hypotheses].

Neanderthals (*Homo neanderthalensis*), our closest extinct relatives, occupy a pivotal position in testing these hypotheses. On the one hand, they share derived traits with our own species *Homo sapiens*, including large-bodied, large-brained neonates, and the corresponding obstetrical challenges (29). On the other hand, Neanderthals retain plesiomorphic features of the pelvis absent in modern humans—such as pronounced iliac flaring and a transverse ellipsoid shape—alongside derived traits that have been hypothesized to reflect adaptations to cold climate [such as generally wide pelvic dimensions relative to stature (30)] and/or species-specific locomotor habits (31).

Comparisons between human and Neanderthal pelvic morphologies thus provide a critical framework for evaluating evolutionary pressures related to obstetrics, bipedal locomotion/stance, pelvic floor stability, and thermoregulation. However, limited and fragmentary fossil material has restricted robust comparative inferences. For example, anatomy-based virtual reconstructions of the adult female Neanderthal pelvis from Tabun (Israel) and the neonate from Mezmaiskaya (Russia) have suggested a rotational birth mechanism analogous to that in modern humans (29). Conversely, a regression-based reconstruction of the Tabun pelvis using a modern human reference sample indicated a transverse birth mode resembling that proposed for *Australopithecus* and early *Homo* (32). Yet the inferred obstetrical dimensions would have been too constricted for the delivery of a Neanderthal neonate (33). Additionally, both male and female Neanderthal pelves have been characterized as transverse ellipsoid in shape (29, 31, 32, 34), leaving open the question of whether PSD in this species aligns with predictions from the human-tailored ODH and/or MPH. Finally, as pelvic morphology undergoes substantial developmental change throughout life (35), it remains unclear which evolutionary developmental processes underpin the differences observed between Neanderthal and modern human pelves.

**Fig. 1.**
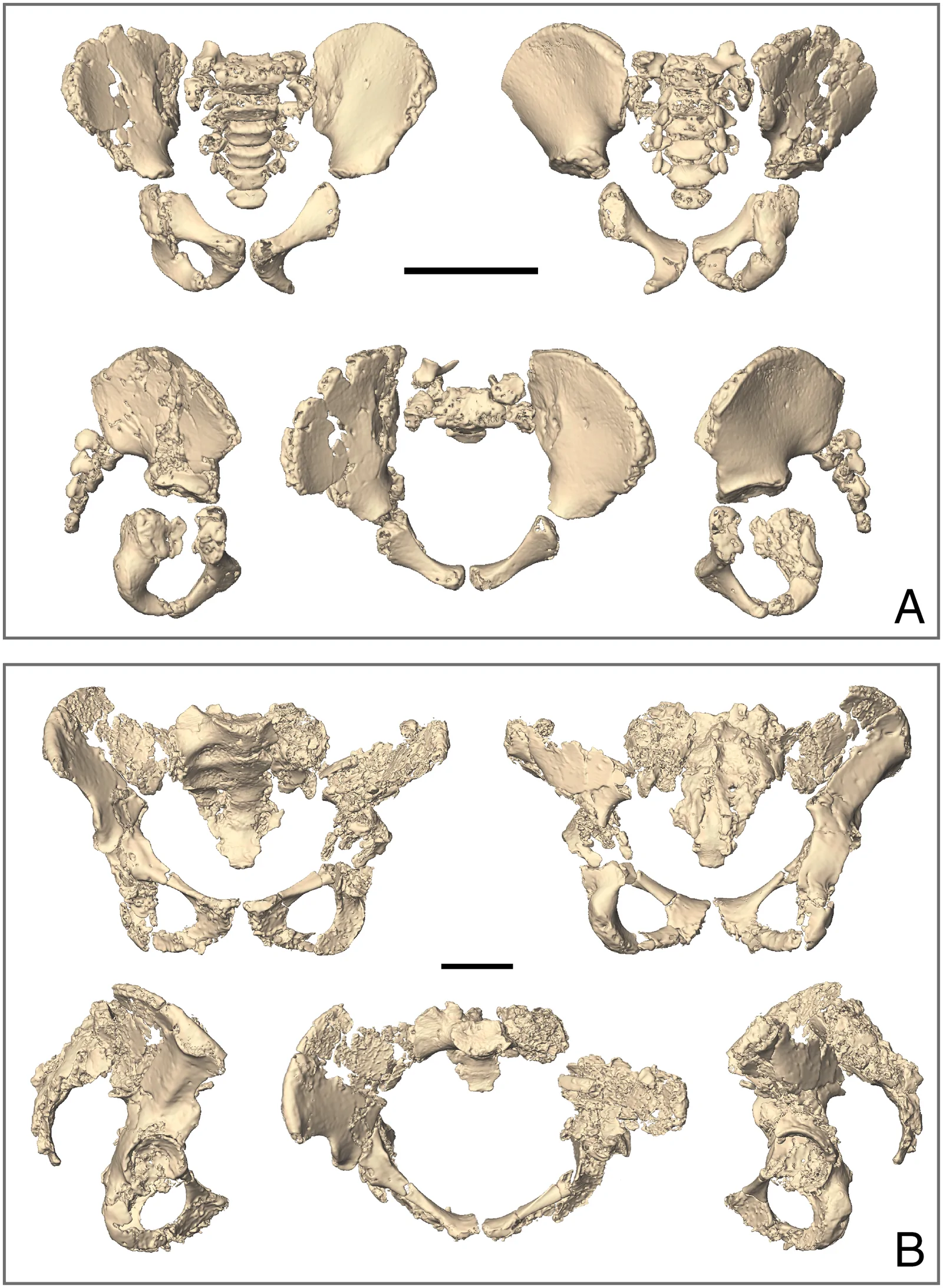
Virtual reconstruction of Neanderthal pelves. (*A*) Infant from Dederiyeh Cave (“Dederiyeh 1”). (*B*) Young adult female from Sima de las Palomas del Cabezo Gordo (“Paloma”). From *Left* to *Right* and *Top* to *Bottom*: Anterior, posterior, *Left* lateral, anterosuperior, and *Right* lateral views. (Scale bar, 5 cm.)

To address these questions, we extend existing data on Neanderthal pelvic morphology (29, 36) by incorporating new 3D reconstructions and morphometric analyses of two nearly complete pelves: one belonging to a 1.5-y-old infant from Dederiyeh Cave (Syria) (37) (Fig. 1*A*) and the other belonging to a young adult female from the Sima de las Palomas del Cabezo Gordo (Murcia, Spain) (38) (Fig. 1*B*).

## Results

The Dederiyeh infant skeleton is well preserved overall, though the pelvic girdle elements remained unfused on account of the individual’s immaturity (39). Its three-dimensional morphology was reconstructed in virtual anatomical space (40) using retrospective clinical CT data of modern human infants to estimate the soft tissue space between the developing bones and by observing structural continuity across the bony elements (Fig. 1*A* and *Materials and Methods* and *SI Appendix*, section 1 and Fig. S1).

The Palomas specimen, recovered from breccia infill in 2006–2007 and subsequently partially prepared, is generally well-preserved but exhibits in situ fragmentation and taphonomic distortion necessitating virtual cleaning and reconstruction (40) prior to analysis (Fig. 1*B* and *SI Appendix*, sections 2 and 3 and Fig. S2). To this end, volumetric data of the fossil were acquired with medical computed tomography (CT). Following digital removal of the sediment matrix, three-dimensional (3D) surface representations of all fossil fragments were generated. These were positioned and oriented in virtual anatomical space using preserved contacts between adjacent parts and observing anatomical constraints, such as bilateral symmetry. Missing regions were complemented with mirror-images of existing counterparts (*SI Appendix*, Fig. S3).

Given their distinct ontogenetic stages, we analyzed the Dederiyeh and Palomas fossils separately, using age-matched comparative samples and measurement protocols for each analysis (*Materials and Methods*). The comparative sample of human infants spans an age range of 1 mo to 8.5 y. The adult comparative sample includes virtual reconstructions of two additional Neanderthal pelves—the Tabun female and Kebara male (29) (*SI Appendix*, section 4 and Table S1)—and a modern human sample comprising individuals aged 14 to 49 y, largely representing the female reproductive life phase. Pelvic morphology was quantified with 3D anatomical points of reference, so-called landmarks (*SI Appendix*, Table S2). The landmark data were then used to perform geometric morphometric analyses of shape variation (*Materials and Methods* and *SI Appendix*, section 5), and to derive linear, angular, and area measurements (*SI Appendix*, Table S3).

**Fig. 2.**
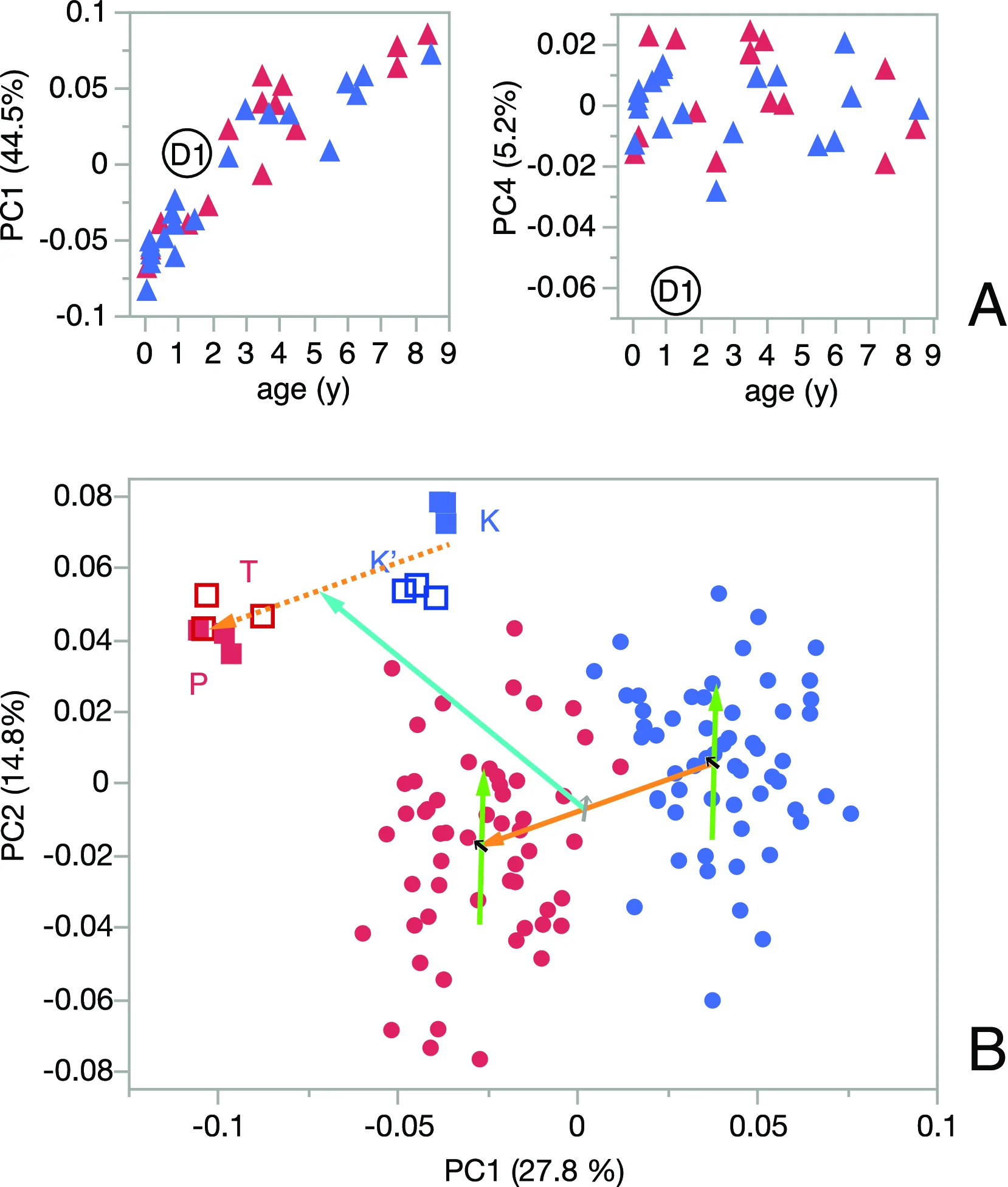
Patterns of Neanderthal and human pelvic shape variation in morphospace. (*A*) Variation in infants. PC1 accounts for 44.5% of total shape variation, mainly due to age-related change in shape (also see *SI Appendix*, Fig. S5). PC4 is independent of age and differentiates the Dederiyeh 1 Neanderthal (D1) from human infants (red/blue triangles: females/males). (*B*) Variation in adult pelvic shape along the first two principal components of shape space, accounting for 27.8% (PC1) and 14.8% (PC2) of total shape variation. Red: females; blue: males; circles: *H. sapiens*; squares: *H. neanderthalensis*; filled squares: Palomas (P) and Kebara (K); open squares: Tabun (T) from ref. 29 and Kebara (K′) from ref. 36. Each fossil specimen is represented by three reconstructive variants. Colored arrows indicate major modes of shape variation; cyan: human mean to Neanderthal mean, orange: male mean to female mean (evaluated for humans, also projected onto the Neanderthal distribution), green: principal mode of shape variation in the pooled human sample (projected on female and male distributions). Small black arrows indicate size-related (allometric) shape variation (corresponding to ±1 SD of centroid size variation) in males and females. A small gray arrow indicates age-related shape variation (corresponding to ±10 years from mean age) in the pooled human sample.

**Fig. 3.**
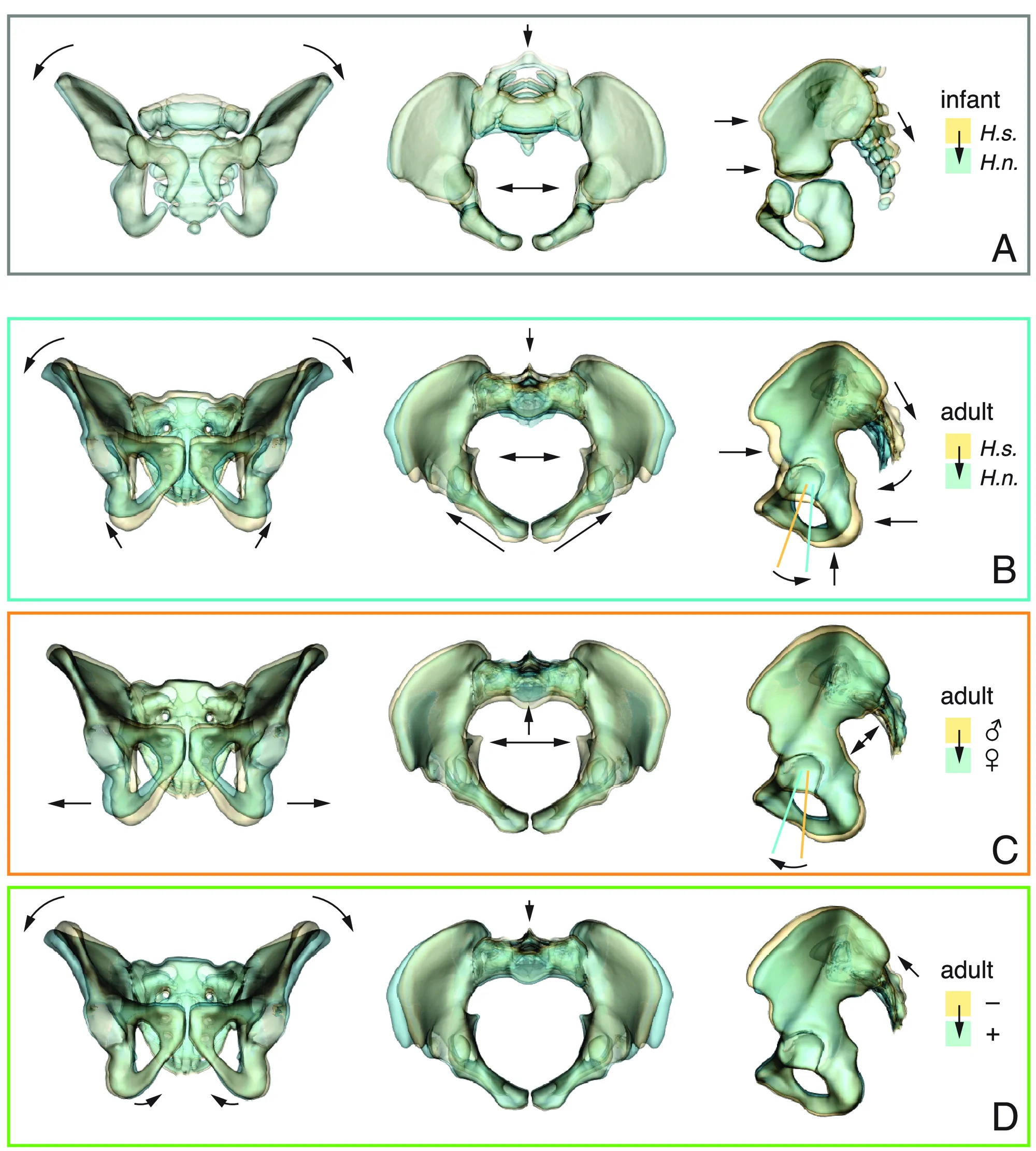
Principal modes of pelvic shape variation. (*A*) Shape differences between human (yellow) and Neanderthal (light blue) infants. (*B*) Shape differences between adult humans (yellow) and Neanderthals (light blue). (*C*) Shape differences between adult males (yellow) and females (light blue). (*D*) Principal mode of adult human shape variation independent of sex (yellow/light blue: ±1 SD). Arrows indicate shape changes from yellow to light-blue morphologies (details see main text).

Pelvic variation in infants is visualized both in multivariate shape space (Fig. 2*A*) and in 3D anatomical space (Fig. 3*A* and *SI Appendix*, section 6 and Fig. S5). The first principal component (PC1) of shape variation (Fig. 2*A*, left graph) captures age-related changes in the shape of the developing pelvis. The 1.5-y-old Dederiyeh 1 Neanderthal infant falls within the upper range of modern human age-related variation in both shape (Fig. 2 *A*, *Left* graph) and size (*SI Appendix*, Fig. S5*A*). Higher-order PCs account for shape variation in the sample regardless of age and size (Fig. 2 *A*, *Right* graph; *SI Appendix*, Fig. S5*B*). Specifically, Dederiyeh 1 is set apart from the modern human sample along PC4 (Fig. 2 *A*, *Right* graph). This mode of shape variation, visualized in Fig. 3*A*, reveals that the Dederiyeh pelvis already had characteristic Neanderthal features distinguishing it from humans, such as flaring iliac blades, a more posterior position of the anterior iliac crest, and a less inclined sacrum, resulting in short anteroposterior relative to transverse dimensions.

Adult pelvic shape variation is visualized in multivariate shape space (Fig. 2*B* and *SI Appendix*, section 7 and Fig. S6) and in 3D anatomical space (Figs. 3 *B*–*D*). Shape space analysis identifies three major modes of variation: interspecific differences between Neanderthals and modern humans (Fig. 3*B*), differences between females and males (Fig. 3*C*), and within-species variation unrelated to sex (Fig. 3*D*). These modes of variation are largely independent of each other (*SI Appendix*, section 7 and Table S4. Pelvic size and individual age account for 2.0% and 3.7% of total shape variation in the modern human sample, respectively (Fig. 2*B*), and are not considered further in this study.

Fig. 3*B* compares adult Neanderthal and modern human average pelvic shapes, pooled across sexes. Neanderthal pelves exhibit wide transverse dimensions and flaring iliac blades, which contrast with comparatively short anteroposterior and superoinferior dimensions. Specifically, the portion of the iliac blade anterior to the iliac pillar appears compressed anteroposteriorly, shifting the anterior iliac crest posteriorly. Concurrently, the acetabulum is positioned farther posteriorly and oriented more posteriorly. The Neanderthal pubic rami are comparatively elongated, as mentioned earlier (31). However, rather than pushing the pubic symphysis anteriorly, the long rami compensate for the more posterior orientation of the acetabular region (41). Additional features of Neanderthal compared to modern human pelves include a wider subpubic region, a more constricted iliac notch, and a less inclined sacrum.

**Fig. 4.**
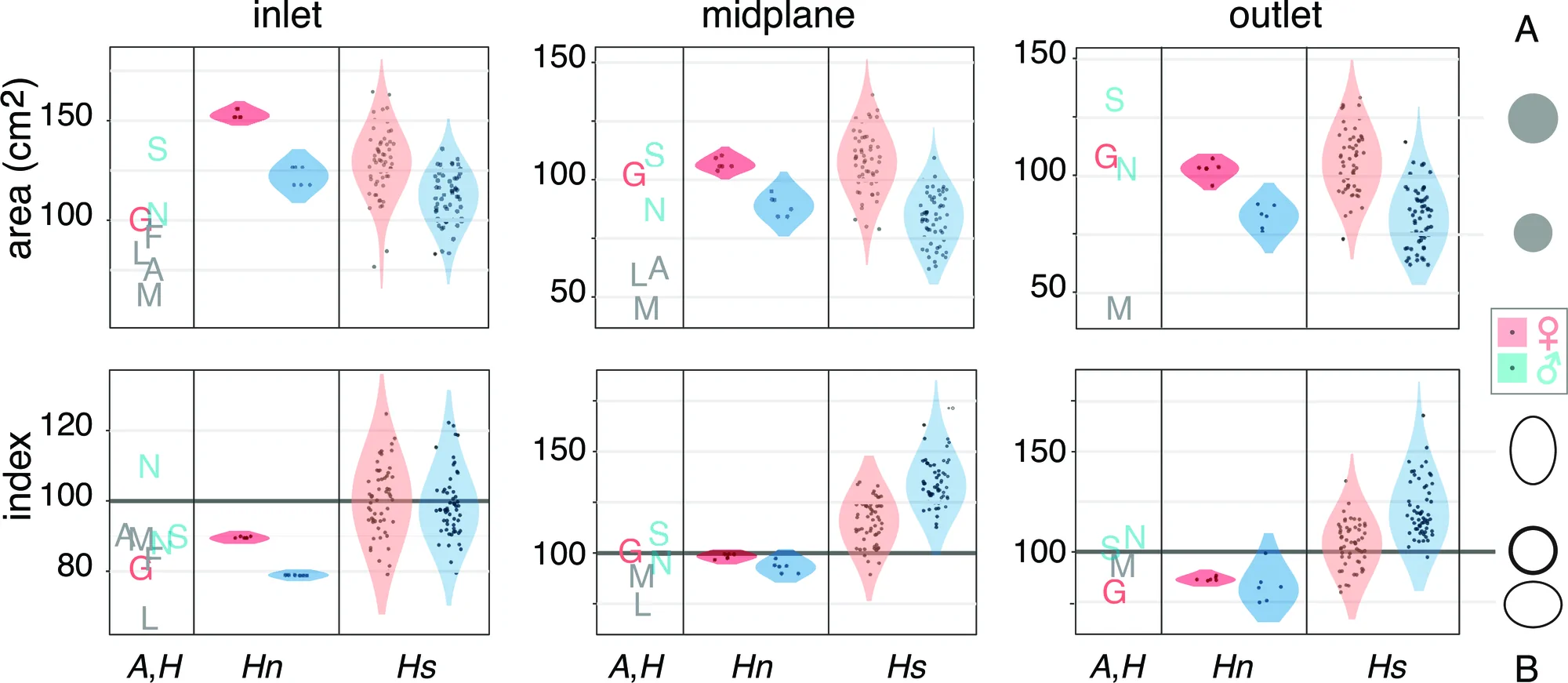
Variation in adult pelvic dimensions related to obstetrics. Inlet, midplane and outlet areas (*A*) and indices (*B*) are measured as products and ratios, respectively, between anteroposterior and transverse dimensions (details see *Materials and Methods* and *SI Appendix*, Table S3). *Hs*: Violin plots for humans (red/blue: female/male). *Hn*: Violin plots for Neanderthals (based on reconstruction variants of Palomas and Tabun females and of Kebara male). *A*,*H*: comparative data from *Australopithecus* and early *Homo*. *A. afarensis* AL288-1 “Lucy” (L), *A. africanus* Sts14 (A), StW573 “Little Foot” (F), *A. sediba* MH2 (M); early *Homo*: Gona (G), Nariokotome (N), Sima de los Huesos SH1 (S).

Pattern and magnitude of sexual dimorphism of the adult Neanderthal pelvis are broadly comparable to modern humans (Fig. 2*B* and *SI Appendix*, section 8 and Fig. S7), reflecting the well-known differences between average female and male morphologies (Fig. 3*C*). Compared to the male pelvis, the female pelvis has wider but inferiorly shorter subpubic and ischial regions, shorter iliac blades (though not more flaring), wider sciatic notches together with a more posteriorly placed sacrum and a more anteriorly oriented acetabulum (Fig. 3*C*). These features result in the large midplane and outlet dimensions that are characteristic of the female pelvis (Fig. 4).

Given the small size of the Neanderthal sample, within-species pelvic variation unrelated to sex could only be assessed in modern humans (Fig. 3*D*). This pattern is best described as rotational variation of the innominate bone in the coronal plane, around the acetabular center: Outward rotation of the iliac blades implies inward rotation of the ischiopubic region, resulting in greater iliac flaring and bi-iliac width but reduced ischiopubic width and concomitant superior shift of the sacrum.

Fig. 4 shows patterns of variation in adult pelvic dimensions that are relevant for obstetrics (*SI Appendix*, section 9 and Fig. S8). Inlet, midplane, and outlet areas—evaluated as the area of ellipses with anteroposterior and transverse anatomical axes (*SI Appendix*, Table S3)—show marked sex-related size dimorphism in both Neanderthals and modern humans (Fig. 4*A*). Human inlet, midplane, and outlet shape indices—evaluated as the ratios of anteroposterior to transverse dimensions—show substantial variation in both sexes, ranging from anteroposteriorly to transversally elongate shapes (Fig. 4*B*). Shape-related sexual dimorphism is present only in the midplane and outlet (although with substantial overlap between sex-specific distributions (42), but not in the inlet (43). The reconstructed female and male Neanderthal pelvic indices are largely similar to each other (Fig. 4), and in the low range of modern human female variation. Notably, the Kebara Neanderthal male indices are outside human male variation.

**Fig. 5.**
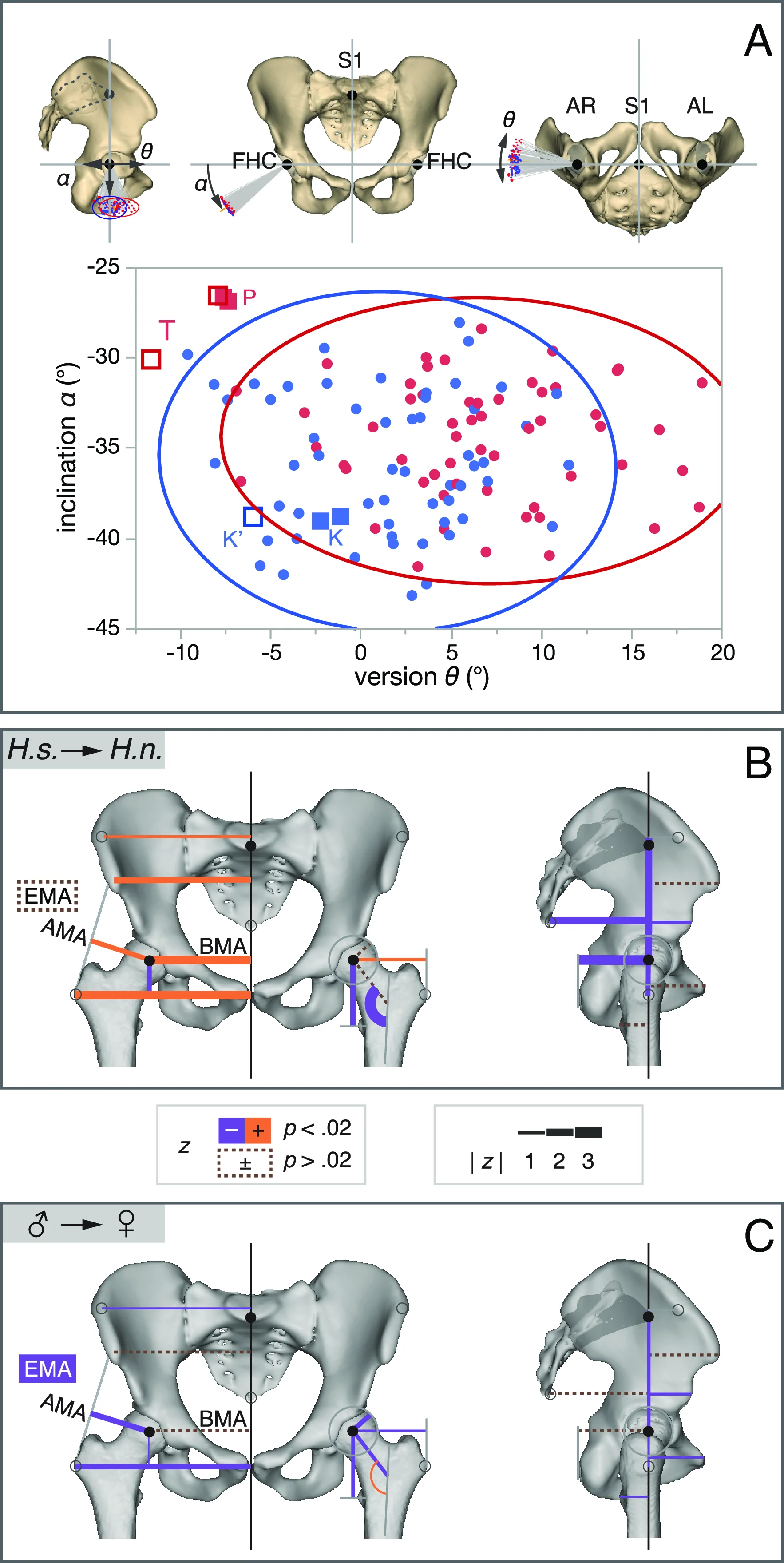
Variation in adult pelvic dimensions related to stance and locomotion. (*A*) Acetabular orientation (inclination *α* and version *θ*) is measured relative to the coronal plane through the centers of the femoral heads (FHC) and the anterosuperior midpoint of the first sacral vertebra (S1). Symbols as in Fig. 2 (see *SI Appendix*, section 10 for details). (*B* and *C*) Mediolateral and anteroposterior distances of pelvic landmarks from the FHC serve as proxies for the moment arm lengths of abductor and flexor/extensor muscles and body weight. (*B*) Size- and sex-standardized contrasts of Neanderthals relative to humans. (*C*) Size-standardized contrasts of human females relative to males. Orange/violet lines indicate significantly longer/shorter distances, respectively, in the first-named group (line thickness is proportional to z-score magnitude); dashed brown lines indicate nonsignificant differences. AMA, abductor moment arm; BMA, body weight moment arm; EMA, effective mechanical advantage (AMA/BMA).

Fig. 5 characterizes adult pelvic shape variation relevant for the biomechanics of stance and locomotion (*SI Appendix*, sections 10 and 11). Fig. 5*A* illustrates variation in acetabular orientation (also see *SI Appendix*, Fig. S9). While modern human females exhibit slightly greater anteversion than males, substantial overlap exists between the sexes. In Neanderthals, the acetabular orientation of both females and males is at the rear end of the modern human distribution.

Fig. 5*B* illustrates differences in Neanderthal and modern human femoropelvic dimensions, which serve as proxies for abductor, flexor/extensor, and body-weight moment arm lengths, adjusted for sex and pelvic centroid size (details in *SI Appendix*, Fig. S10 and Table S5). Compared to modern humans, Neanderthals exhibit consistently wider mediolateral dimensions, leading to longer abductor and body-weight moment arms. However, the relatively longer Neanderthal body-weight moment arm (BMA) is balanced by a correspondingly longer abductor moment arm (AMA). This is driven primarily by a low femoral head-neck angle, which positions the greater trochanter more laterally relative to the femoral head center (FHC). Consequently, this morphology yields an effective mechanical advantage (EMA = AMA/BMA; Fig. 5*B*) of the hip abductors comparable to that of modern humans. Other biomechanically relevant dimensions differ significantly between species: in Neanderthals, the anteroposteriorly short ischium, the more anteriorly positioned sacrum, and the closer proximity of the anterior inferior iliac spine and lesser trochanter to the FHC result in shorter moment arms for both hip extensors and flexors.

Fig. 5*C* illustrates size-adjusted differences in biomechanical features between modern human female and male pelves (*SI Appendix*, Table S5). Compared to interspecific contrasts (Fig. 5*B*), sex-related differences are less pronounced (*SI Appendix*, Table S5). Most notably, females and males exhibit similar lateral distances from the FHC to the body’s midsagittal plane, indicating comparable body-weight moment arm (BMA) lengths for a given pelvic size. However, relative to males, females exhibit larger femoral head-neck angles, smaller femoral heads, and reduced lateral distances of the greater trochanter and the iliac tubercle. Collectively, this geometry results in a lower abductor EMA in females compared to males (3). Additionally, females have relatively shorter flexor-related anteroposterior pelvic dimensions.

Correlation of the Neanderthal–modern human (Fig. 5*B*) and female–male (Fig. 5*C*) contrasts indicate that interspecific and sex-specific biomechanical differences are independent of each other, representing two distinct biological signals (*R*^2^ = 0.002, *P* = 0.54; see *SI Appendix*, Table S5 and Fig. S10).

## Discussion

The expanded Neanderthal pelvic sample offers a broader comparative framework for investigating the evolutionary and developmental processes that shaped the characteristic Neanderthal and modern human pelvic morphologies.

### Evolutionary and Developmental Divergence of Neanderthal and Modern Human Pelvic Morphologies.

At an estimated age of death of 1.5 y, the Dederiyeh infant already exhibited key features of the Neanderthal pelvic architecture—anteroposteriorly short compared to transversely wide dimensions (Fig. 3*A*)—indicating that these traits emerged early during ontogeny (Fig. 3*A* and *SI Appendix*, section 6 and Fig. S5). This complements the observation that the linear pelvic dimensions of the Neanderthal neonates from Mezmaiskaya and Le Moustier are at the fringe of human neonate variation (29, 44), and aligns with developmental evidence suggesting that species-specific pelvic form is established through divergent prenatal growth trajectories reflecting differences in localized regulation of growth (45). In modern humans, for example, the unique anterior expansion of the iliac blade is facilitated by an extended period of chondrogenesis and delayed ossification (46), a developmental mode likely less expressed in the Neanderthals with their less projecting anterior iliac crests (Figs. 3 *A* and *B*).

Our observation of fairly independent patterns of pelvic shape variation between species, between sexes, and within species (Figs. 2 and 3 and *SI Appendix*, Table S4) is in line with earlier studies showing that the hominin pelvis exhibits a high degree of modularity, allowing different regions to develop and evolve fairly independently (47–49). These findings support the view that the evolutionary divergence of modern human and Neanderthal pelvic morphologies involved species-specific shifts in local growth rates and timing (heterochrony), and module-specific regulatory changes (45).

### Pelvic Morphology and Obstetrics.

The presence of a shared pattern of sexual dimorphism in the otherwise clearly distinct Neanderthal and human pelvic morphologies provides a unique opportunity to assess tradeoffs between obstetrical, locomotor, and other constraints. The larger female than male sizes of the three obstetrical planes—inlet, midplane, and outlet—likely reflect the shared constraint of bearing and birthing large-brained large-bodied babies.

The possible association between birth canal shape, birth mechanisms, and obstetrical complications was first explored in two seminal studies (50, 51), which have since undergone extensive reevaluation (52). Our data show that pelvic inlet shape—unlike inlet size—exhibits no sexual dimorphism (Fig. 4 and *SI Appendix*, Fig. S8) (43) and is therefore unlikely to have been subject to obstetrical selection. Moreover, an unbiased reanalysis of the original data presented in refs. 50 and 51 does not support a statistically robust correlation between pelvic shape and birth complications (*SI Appendix*, section 12 and Table S6). Furthermore, independent of intrapopulation pelvic shape variation, fetal head orientation during delivery shows variation along the occiput-anterior/posterior spectrum, with the lowest incidence of birth complications occurring in occiput-anterior presentation combined with favorable fetopelvic size proportions (53). This suggests that inferences about obstetrical mechanisms drawn from fossil or modern pelvic skeletal shapes alone (29) and/or using a typology-oriented approach (32, 50, 51) should be treated with caution, as they risk underestimating the extent of natural variation in actual birth mechanisms, and the influence of soft tissue-related constraints on the birth process (28).

Another hypothesis holds that the morphology of the lower birth canal (midplane and outlet) reflects adaptations for pelvic floor stability driven by bipedalism (7, 35). Biomechanical models indicate that, for a given canal area, anteroposteriorly elongate shapes better resist floor deformation than more rounded shapes (54). However, various observations suggest that female pelvic floor stability (i.e., the ability to resist floor deformation) in humans is more likely constrained by the size of the birth canal (7)—and correspondingly that of the term fetus—than by birth canal shape. Midplane and outlet shapes range widely—from anteroposteriorly to transversely elongated forms (Fig. 4 and *SI Appendix*, Fig. S8) (42)—and females tend to exhibit more rounded canal shapes than males (Fig. 4). Also, female midplane and outlet shapes do not correlate with canal size (*SI Appendix*, Fig. S8), reinforcing the notion that size, rather than shape, plays a critical role in maintaining pelvic floor stability. Against this comparative background, the Neanderthal birth canal, with its wide transverse and short anteroposterior dimensions, but human-like sex-specific size dimorphism (Fig. 4 and *SI Appendix*, section 9), appears to reflect similar evolutionary tradeoffs between fetopelvic proportion and pelvic floor stability as in modern humans. Overall, it thus appears that the obstetrical dilemma is between obstetrics (i.e., accommodating a large fetal head) and maintaining pelvic floor stability, rather than between obstetrics and bipedal efficiency, and that it is more likely that birth canal size (55) rather than shape was the major evolutionary target.

### Pelvic Morphology, Obstetrics, and Locomotion.

Contrary to the expectations of the obstetrical dilemma hypothesis (ODH) (1), the wider birth canal dimensions of female pelves (Fig. 4) do not result in a wider distance between the hip joints and longer body-weight moment arms (Fig. 5*C*). Rather than increasing the FHC distance, wide transverse obstetric dimensions are achieved through internal architectural shifts, including smaller femoral heads (Fig. 5*C* and *SI Appendix*, section 13 and Fig. S11) and a more slender iliac isthmus (35). Consequently, the lower abductor EMA observed in females (3) is an effect of reduced lateral distance of the greater trochanter rather than a longer body-weight lever arm (Fig. 5*C*). The functional implications of this configuration remain to be elucidated further, especially concerning sex-related differences in body size and leg length, and in the light of experimental and model evidence that variation in abductor EMA has negligible influence on total locomotor cost (4–6, 56).

### Species-Specific Pelvic Morphology and Locomotion.

Our data further indicate that Neanderthal-modern human differences in femoropelvic biomechanical properties are not related to the sex-specific biomechanical constraints discussed before (Fig. 5 and *SI Appendix*, Fig. S10*B*) and can therefore be considered independently. Neanderthals exhibit acetabular version at the posterior end of modern human variation (Fig. 5*A*). Since acetabular version correlates with pelvic tilt (57), which in turn correlates with lumbar lordosis during movement (58–60), we hypothesize that Neanderthals exhibited comparatively less tilt and lordosis than modern-day humans, as proposed earlier (61, 62) [but see (63) and *SI Appendix*, Fig. S9]. In this regard, Neanderthals likely more closely resembled preindustrial human populations (64) than modern-day samples. Furthermore, while Neanderthals and modern humans exhibited similar hip abductor EMA to balance the body during single-leg stance (Fig. 5*B*), Neanderthals exhibited markedly shorter lever arms for both hip extensors and flexors. Together with reduced pelvic tilt and lumbar lordosis, this architecture likely prioritized trunk stability and power stride generation over endurance running capabilities (25, 26, 65–68).

Overall, the Neanderthal architecture may have incurred higher metabolic costs of locomotion than the modern human architecture, as shorter lever arms require higher muscle force and/or recruitment frequencies to move the body at a given speed. However, inferences on metabolic effectiveness must remain preliminary. The lever arm models applied here are essentially static (Fig. 5), while dynamic adjustments of muscle activity tend to optimize costs of locomotion (3, 5, 6). Furthermore, the use of human-based physiological and biomechanical modeling parameters inherently limits interspecific inferential power (5). Ultimately, how this unique lever geometry influenced locomotor patterns and efficiency must be tested through dynamic, individualized whole-body simulations including further Neanderthal-human contrasts in limb length and proportions (6, 69).

### Pelvic Morphology and Thermoregulation.

Beyond locomotion, the Neanderthal pelvis is frequently interpreted as exhibiting cold-climate adaptations for heat conservation (70). However, by expanding Ruff’s traditional cylindrical body model (71) to a cylindroid model that accounts for different anteroposterior and transverse dimensions (*SI Appendix*, section 14), we find that Neanderthal pelvic surface-to-volume (S/V) ratios fall well within the range of modern human variation (*SI Appendix*, Fig. S12). While cold adaptation may be reflected in other skeletal regions, such as the thorax (70), the pelvic morphology of the Neanderthals studied here—all of whom inhabited temperate paleoclimates—does not support such a thermoregulatory signal. This aligns with evidence that local climate has a negligible effect on modern human pelvic shape once population history is taken into account (72).

### Behavioral Implications.

Ancient DNA analyses indicate several periods of interaction and hybridization between Neanderthal and early modern human populations, spanning several millennia and leaving enduring genomic traces in present-day humans (73–75). Such genetic exchange necessarily entailed Neanderthal and/or human mothers giving birth to hybrid offspring (76). The shared Neanderthal-human obstetrical evolutionary history inferred here may have influenced biomechanical or developmental factors relevant to parturition in these mixed-ancestry births.

### Conclusion.

Overall, the evidence from Palomas and Dederiyeh accentuates the contrasting pelvic morphologies of Neanderthals and modern humans. Data from *Australopithecus* and *Homo* pelves predating the Neanderthals (Fig. 4, letter symbols) indicate that the transverse ellipsoid morphology of the pelvis is a symplesiomorphic feature of hominins, probably further accentuated during Neanderthal evolution, while the anteroposterior ellipsoid morphology of modern humans represents a derived state (77, 78). Our data further suggest that these morphologies are not necessarily linked to obstetrical constraints, as an earlier study (31) also suggested. It remains to be investigated to what extent the species-specific features of Neanderthal and human pelves reflect adaptive evolution of different locomotor modes (31, 79) and to what extent they reflect neutral divergence (30).

## Materials and Methods

### Neanderthal Sample.

The infant sample comprises the uniquely well preserved pelvis of the 1.5-y-old Dederiyeh 1 specimen (37). The adult sample comprises the female specimens from Palomas (this study) and Tabun (29), and the male specimen from Kebara (29). In morphometric analyses, each adult specimen is represented by three reconstructive variants. Step-by-step protocols of the Neanderthal pelvic reconstructions are provided in the *SI Appendix*, sections 1–4. An independent virtual reconstruction of Kebara (36) was also included in the adult sample.

### Comparative Modern Human Samples.

The human pelvis develops from birth to adulthood along a multiphase nonlinear trajectory (35). To manage the resulting complexities of Neanderthal-human comparisons at different developmental stages, we performed two independent morphometric analyses, one for infants, the other for adults. The comparative human infant sample consists of anonymized retrospective clinical CT data from ref. 35 of *N*_i_ = 34 individuals aged 1 mo to 8.4 y (15 females, 19 males). The comparative adult human sample consists of anonymized retrospective clinical CT data from ref. 35 (all without ostensive skeletal pathologies) of *N*_a_ = 112 individuals (56 females, 56 males) aged 14 to 49 y. Adult pelvic morphologies were quantified with *K*_a_ = 123 3D anatomical points of reference (landmarks), infant morphologies with *K*_i_ = 153 3D landmarks. The larger number of landmarks in the infant sample reflects anatomical reference points on unfused bony elements that converge in the fully ossified adult pelvis. Details see *SI Appendix*, sections 5–7.

### Morphometric Analyses.

Geometric morphometric (GM) analyses (80) of shape variation were performed separately for the adult and the infant samples, using the respective landmark datasets. Because natural patterns of bilateral asymmetry cannot be reliably reconstructed in fossil specimens, all landmark configurations were symmetrized. Generalized Procrustes Analysis was performed on the size-standardized landmark configurations. Patterns of shape variation in the sample were explored with principal components analysis (PCA) of shape. Pelvic size (quantified as centroid size) was used as an independent external variable to study its effects on shape. All analyses were performed with the *R* packages Geomorph (81), Morpho, and Rvcg (82). Details see *SI Appendix*, sections 6 and 7.

## Supplementary Material

Appendix 01 (PDF)

Dataset S01 (XLSX)

Dataset S02 (XLSX)

## Data Availability

All study data are included in the article and in supporting information and Datasets S1 and S2.

## References

[1] S. L. Washburn, Tools and human evolution. Sci. Am. **203**, 63–75 (1960). 13843002

[2] H. M. Dunsworth, A. G. Warrener, T. Deacon, P. T. Ellison, H. Pontzer, Metabolic hypothesis for human altriciality. Proc. Natl. Acad. Sci. U.S.A. **109**, 15212–15216 (2012). 22932870 10.1073/pnas.1205282109 PMC3458333

[3] A. G. Warrener, K. L. Lewton, H. Pontzer, D. E. Lieberman, A wider pelvis does not increase locomotor cost in humans, with implications for the evolution of childbirth. PLoS ONE **10**, e0118903 (2015). 25760381 10.1371/journal.pone.0118903 PMC4356512

[4] C. M. Wall-Scheffler, M. J. Myers, The biomechanical and energetic advantages of a mediolaterally wide pelvis in women. Anat. Rec. **300**, 764–775 (2017). 10.1002/ar.23553 28297181

[5] P. A. Kramer, A. D. Sylvester, Hip width and metabolic energy expenditure of abductor muscles. PLoS ONE **18**, e0284450 (2023). 37071649 10.1371/journal.pone.0284450 PMC10112780

[6] E. Stansfield, C. Egner, P. Mitteroecker, H. Kainz, Did energy costs of walking limit the evolution of a larger human birth canal? Proc. R. Soc. B Biol. Sci. **293**, 20252895 (2026). 10.1098/rspb.2025.2895 41667102

[7] E. Stansfield, K. Kumar, P. Mitteroecker, N. D. S. Grunstra, Biomechanical trade-offs in the pelvic floor constrain the evolution of the human birth canal. Proc. Natl. Acad. Sci. U.S.A. **118**, e2022159118. (2021). 33853947 10.1073/pnas.2022159118 PMC8072325

[8] P. Mitteroecker, S. M. Huttegger, B. Fischer, M. Pavlicev, Cliff-edge model of obstetric selection in humans. Proc. Natl. Acad. Sci. U.S.A. **113**, 14680–14685 (2016). 27930310 10.1073/pnas.1612410113 PMC5187675

[9] L. Xu , The genetic architecture of and evolutionary constraints on the human pelvic form. Science **388**, eadq1521 (2025). 40208988 10.1126/science.adq1521

[10] A. Warrener, The multifactor pelvis: An alternative to the adaptationist approach of the obstetrical dilemma. Evol. Anthropol. **32**, 260–274 (2023). 37527355 10.1002/evan.21997

[11] M. Haeusler , The obstetrical dilemma hypothesis: There’s life in the old dog yet. Biol. Rev. **96**, 2031–2057 (2021). 34013651 10.1111/brv.12744 PMC8518115

[12] N. D. S. Grunstra , There is an obstetrical dilemma: Misconceptions about the evolution of human childbirth and pelvic form. Am. J. Biol. Anthropol. **181**, 535–544 (2023). 37353889 10.1002/ajpa.24802 PMC10952510

[13] A. Huseynov, Comparative Ontogeny of the Hominid Pelvis and Implications for the Evolution of Birth (University of Zurich, 2016).

[14] B. Fischer, N. D. S. Grunstra, E. Zaffarini, P. Mitteroecker, Sex differences in the pelvis did not evolve de novo in modern humans. Nat. Ecol. Evol. **5**, 625–630 (2021). 33767411 10.1038/s41559-021-01425-z PMC7617076

[15] W. Leutenegger, “Encephalization and obstetrics in primates, with particular reference to human evolution” in Primate Brain Evolution; Methods and Concepts, E. Armstrong, D. Falk, Eds. (Plenum Press, 1982), pp. 85–96.

[16] M. S. Ponce de León, C. P. E. Zollikofer, “Primate birth at the extremes: Exploring obstetric and metabolic constraints” in Costly and Cute: Helpless Infants and Human Evolution, W. Trevathan, K. Rosenberg, Eds. (SAR Press, 2016), pp. 51–66.

[17] M. Kawada, M. Nakatsukasa, T. Nishimura, A. Kaneko, N. Morimoto, Covariation of fetal skull and maternal pelvis during the perinatal period in rhesus macaques and evolution of childbirth in primates. Proc. Natl. Acad. Sci. U.S.A. **117**, 21251–21257 (2020). 32817513 10.1073/pnas.2002112117 PMC7474677

[18] N. M. Laudicina, M. Cartmill, Bony birth-canal dimensions and obstetric constraints in hominoids. Am. J. Biol. Anthropol. **180**, 442–452 (2023).

[19] T. D. White , *Ardipithecus ramidus* and the paleobiology of early hominids. Science **326**, 75–86 (2009). 19810190

[20] W. Leutenegger, Newborn size and pelvic dimensions of *Australopithecus*. Nature **240**, 568–569 (1972). 4568405 10.1038/240568a0

[21] C. O. Lovejoy, The obstetric pelvis of AL-288-1 (Lucy). J. Hum. Evol. **15**, 237–255 (1986).

[22] R. G. Tague, C. O. Lovejoy, AL 288–1—Lucy or Lucifer: Gender confusion in the Pliocene. J. Hum. Evol. **35**, 75–94 (1998). 9680468 10.1006/jhev.1998.0223

[23] J. M. DeSilva, A shift toward birthing relatively large infants early in human evolution. Proc. Natl. Acad. Sci. U.S.A. **108**, 1022–1027 (2011). 21199942 10.1073/pnas.1003865108 PMC3024680

[24] K. R. Rosenberg, The evolution of modern human childbirth. Am. J. Phys. Anthropol. **35**, 89–124 (1992).

[25] C. B. Ruff, Biomechanics of the hip and birth in early *Homo*. Am. J. Phys. Anthropol. **98**, 527–574 (1995). 8599386 10.1002/ajpa.1330980412

[26] D. M. Bramble, D. E. Lieberman, Endurance running and the evolution of *Homo*. Nature **432**, 345–352 (2004). 15549097 10.1038/nature03052

[27] C. Berge, D. Goularas, A new reconstruction of Sts 14 pelvis (*Australopithecus africanus*) from computed tomography and three-dimensional modeling techniques. J. Hum. Evol. **58**, 262–272 (2010). 20138331 10.1016/j.jhevol.2009.11.006

[28] P. Frémondière , Dynamic finite-element simulations reveal early origin of complex human birth pattern. Commun. Biol. **5**, 377 (2022). 35440693 10.1038/s42003-022-03321-z PMC9018746

[29] M. S. Ponce de León , Neanderthal brain size at birth provides insights into the evolution of human life history. Proc. Natl. Acad. Sci. U.S.A. **105**, 13764–13768 (2008). 18779579 10.1073/pnas.0803917105 PMC2533682

[30] T. D. Weaver, The meaning of Neandertal skeletal morphology. Proc. Natl. Acad. Sci. U.S.A. **106**, 16028–16033 (2009). 19805258 10.1073/pnas.0903864106 PMC2752516

[31] Y. Rak, B. Arensburg, Kebara 2 Neanderthal pelvis: First look at a complete inlet. Am. J. Phys. Anthropol. **73**, 227–231 (1987). 3113264 10.1002/ajpa.1330730209

[32] T. D. Weaver, J. J. Hublin, Neandertal birth canal shape and the evolution of human childbirth. Proc. Natl. Acad. Sci. U.S.A. **106**, 8151–8156 (2009). 19380728 10.1073/pnas.0812554106 PMC2688883

[33] C. P. E. Zollikofer, M. S. Ponce de León, Reconsidering Neanderthal birth: Tabun mom, Kebara dad, Mezmaiskaya baby, and the consequences. Amercian J. Phys. Anthropol. **141**, 252 (2010).

[34] Y. Rak, On the differences between two pelvises of Mousterian context from the Qafzeh and Kebara caves, Israel. Am. J. Phys. Anthropol. **81**, 323–332 (1990). 2327476 10.1002/ajpa.1330810302

[35] A. Huseynov , Developmental evidence for obstetric adaptation of the human female pelvis. Proc. Natl. Acad. Sci. U.S.A. **113**, 5227–5232 (2016). 27114515 10.1073/pnas.1517085113 PMC4868434

[36] M. T. Adegboyega, P. A. Stamos, J. J. Hublin, T. D. Weaver, Virtual reconstruction of the Kebara 2 Neanderthal pelvis. J. Hum. Evol. **151**, 102922 (2021). 33360685 10.1016/j.jhevol.2020.102922

[37] T. Akazawa, S. Muhesen, Y. Dodo, O. Kondo, Y. Mizoguchi, Neanderthal infant burial. Nature **377**, 585–586 (1995). 7566170 10.1038/377585a0

[38] M. J. Walker, J. Ortega, K. Parmová, M. V. López, E. Trinkaus, Morphology, body proportions, and postcranial hypertrophy of a female Neandertal from the Sima de las Palomas, southeastern Spain. Proc. Natl. Acad. Sci. U.S.A. **108**, 10087–10091 (2011). 21646528 10.1073/pnas.1107318108 PMC3121844

[39] O. Kondo, Y. Dodo, T. Akazawa, S. Muhesen, Estimation of stature from the skeletal reconstruction of an immature Neandertal from Dederiyeh Cave, Syria. J. Hum. Evol. **38**, 457–473 (2000). 10715192 10.1006/jhev.1999.0347

[40] C. P. E. Zollikofer, M. S. Ponce de León, Virtual Reconstruction: A Primer in Computer-Assisted Paleontology and Biomedicine (Wiley, 2005).

[41] G. S. Krantz, Evolution of the human pelvis. Evol. Theory Rev. **11**, 255–272 (1997).

[42] L. Betti, Shaping birth: Variation in the birth canal and the importance of inclusive obstetric care. Philos. Trans. R. Soc. B Biol. Sci. **376**, 20200024 (2021). 10.1098/rstb.2020.0024 PMC8090820 33938285

[43] H. Delprete, Pelvic inlet shape is not as dimorphic as previously suggested. Anat. Rec. **300**, 706–715 (2017). 10.1002/ar.23544 28297189

[44] T. D. Weaver , Neonatal postcrania from Mezmaiskaya, Russia, and Le Moustier, France, and the development of Neandertal body form. Proc. Natl. Acad. Sci. U.S.A. **113**, 6472–6477 (2016). 27217565 10.1073/pnas.1523677113 PMC4988559

[45] M. Young , The developmental impacts of natural selection on human pelvic morphology. Sci. Adv. **8**, 4884 (2022). 10.1126/sciadv.abq4884 PMC9385149 35977020

[46] D. Zirkle, C. O. Lovejoy, The hominid ilium is shaped by a synapomorphic growth mechanism that is unique within primates. Proc. Natl. Acad. Sci. U.S.A. **116**, 13915–13920 (2019). 31235562 10.1073/pnas.1905242116 PMC6628783

[47] M. W. Grabowski, J. D. Polk, C. C. Roseman, Divergent patterns of integration and reduced constraint in the human hip and the origins of bipedalism. Evolution (N.Y.) **65**, 1336–1356 (2011). 10.1111/j.1558-5646.2011.01226.x 21521191

[48] M. Grabowski, C. C. Roseman, Complex and changing patterns of natural selection explain the evolution of the human hip. J. Hum. Evol. **85**, 94–110 (2015). 26164108 10.1016/j.jhevol.2015.05.008

[49] M. MacKinnon, L. Harrington, L. W. Cowgill, H. K. Kurki, Ilium morphological variation during growth in forager populations. J. Hum. Evol. **205**, 103717 (2025). 40592052 10.1016/j.jhevol.2025.103717

[50] H. Thoms, Obstetrical significance of pelvic variations. Br. Med. J. **2**, 210 (1937). 20780813 10.1136/bmj.2.3995.210 PMC2087011

[51] W. E. Caldwell, H. C. Moloy, Anatomical variations in the female pelvis: Their classification and obstetrical significance. Proc. R. Soc. Med. **32**, 1–30 (1938). 19991699 10.1177/003591573803200101 PMC1997320

[52] C. VanSickle, K. L. Liese, J. N. Rutherford, Textbook typologies: Challenging the myth of the perfect obstetric pelvis. Anat. Rec. **305**, 952–967 (2022). 10.1002/ar.24880 PMC9303659 35202515

[53] K. Bagandanshwa , Fetal rotation examined with ultrasound in a sub-Saharan population: A longitudinal cohort study. Acta Obstet. Gynecol. Scand. **104**, 225–234 (2025). 39568051 10.1111/aogs.15013 PMC11683555

[54] E. Stansfield, B. Fischer, N. D. S. Grunstra, M. V. Pouca, P. Mitteroecker, The evolution of pelvic canal shape and rotational birth in humans. BMC Biol. **19**, 1–11 (2021). 34635119 10.1186/s12915-021-01150-w PMC8507337

[55] T. Starrach , Pelvic inlet area is associated with birth mode. Acta Obstet. Gynecol. Scand. **102**, 59–66 (2023). 36320156 10.1111/aogs.14478 PMC9780724

[56] A. G. Warrener, Hominin hip biomechanics: Changing perspectives. Anat. Rec. **300**, 932–945 (2017). 10.1002/ar.23558 28406571

[57] R. K. Zahn , Pelvic tilt compensates for increased acetabular anteversion. Int. Orthop. **40**, 1571–1575 (2016). 26318879 10.1007/s00264-015-2949-6

[58] J. W. Day, G. L. Smidt, T. Lehmann, Effect of pelvic tilt on standing posture. Phys. Ther. **64**, 510–516 (1984). 6231648 10.1093/ptj/64.4.510

[59] D. Levine, M. W. Whittle, The effects of pelvic movement on lumbar lordosis in the standing position. J. Orthop. Sports Phys. Ther. **24**, 130–135 (1996). 8866271 10.2519/jospt.1996.24.3.130

[60] C. Boulay , Anatomical reliability of two fundamental radiological and clinical pelvic parameters: Incidence and thickness. Eur. J. Orthop. Surg. Traumatol. **15**, 197–204 (2005).

[61] A. Bonmatí , Middle Pleistocene lower back and pelvis from an aged human individual from the Sima de los Huesos site, Spain. Proc. Natl. Acad. Sci. U.S.A. **107**, 18386–18391 (2010). 20937858 10.1073/pnas.1012131107 PMC2973007

[62] E. Been, A. Gómez-Olivencia, P. A. Kramer, A. Barash, “3D reconstruction of spinal posture of the Kebara 2 Neanderthal” in Vertebrate Paleobiology and Paleoanthropology (Springer, Cham, 2017), pp. 239–251.

[63] M. Haeusler , Morphology, pathology, and the vertebral posture of the La Chapelle-aux-Saints Neandertal. Proc. Natl. Acad. Sci. U.S.A. **116**, 4923–4927 (2019). 30804177 10.1073/pnas.1820745116 PMC6421410

[64] S. A. Williams , Inferring lumbar lordosis in Neandertals and other hominins. PNAS Nexus **1**, pgab005 (2022). 36712807 10.1093/pnasnexus/pgab005 PMC9801964

[65] T. D. Weaver, The shape of the Neandertal femur is primarily the consequence of a hyperpolar body form. Proc. Natl. Acad. Sci. U.S.A. **100**, 699–6926 (2003). 10.1073/pnas.1232340100 PMC165806 12761384

[66] C. M. Wall-Scheffler, Energetics, locomotion, and female reproduction: Implications for human evolution. Annu. Rev. Anthropol. **41**, 71–85 (2012).

[67] L. T. Gruss, D. Schmitt, The evolution of the human pelvis: Changing adaptations to bipedalism, obstetrics and thermoregulation. Philos. Trans. R. Soc. B Biol. Sci. **370**, 20140063 (2015). 10.1098/rstb.2014.0063 PMC4305164 25602067

[68] L. T. Gruss, R. Gruss, D. Schmitt, Pelvic breadth and locomotor kinematics in human evolution. Anat. Rec. **300**, 739–751 (2017). 10.1002/ar.23550 PMC6560644 28297175

[69] R. W. Higgins, C. B. Ruff, The effects of distal limb segment shortening on locomotor efficiency in sloped terrain: Implications for Neandertal locomotor behavior. Am. J. Phys. Anthropol. **146**, 336–345 (2011). 22102995 10.1002/ajpa.21575

[70] T. W. Holliday, C. Ocobock, L. W. Cowgill, S. D. Maddux, Neandertal cold adaptation: Technological, anatomical, and physiological responses to cold stress in one of our closest fossil relatives. Am. J. Hum. Biol. **37**, e70150 (2025). 41029952 10.1002/ajhb.70150 PMC12485224

[71] C. B. Ruff, Climate and body shape in hominid evolution. J. Hum. Evol. **21**, 81–104 (1991).

[72] L. Betti, Human variation in pelvic shape and the effects of climate and past population history. Anat. Rec. **300**, 687–697 (2017). 10.1002/ar.23542 28297180

[73] L. Li, T. J. Comi, R. F. Bierman, J. M. Akey, Recurrent gene flow between Neanderthals and modern humans over the past 200,000 years. Science **385**, eadi1768 (2024). 38991054 10.1126/science.adi1768 PMC12885488

[74] L. N. M. Iasi , Neanderthal ancestry through time: Insights from genomes of ancient and present-day humans. Science **386**, 593955 (2024). 10.1126/science.adq3010 PMC12184710 39666853

[75] A. P. Sümer , Earliest modern human genomes constrain timing of Neanderthal admixture. Nature **638**, 711–717 (2024). 39667410 10.1038/s41586-024-08420-x PMC11839475

[76] A. Platt, D. N. Harris, S. A. Tishkoff, Interbreeding between Neanderthals and modern humans was strongly sex biased. Science **391**, 922–925 (2026). 41747031 10.1126/science.aea6774 PMC13345703

[77] C. B. Ruff, Body size and body shape in early hominins - Implications of the Gona pelvis. J. Hum. Evol. **58**, 166–178 (2010). 19945140 10.1016/j.jhevol.2009.10.003

[78] C. M. Wall-Scheffler, H. K. Kurki, B. M. Auerbach, “Pelves of the hominin lineage” in The Evolutionary Biology of the Human Pelvis, C. M. Wall-Scheffler, H. K. Kurki, B. M. Auerbach, Eds. (Cambridge University Press, 2020), pp. 46–98.

[79] M. W. Grabowski, Hominin obstetrics and the evolution of constraints. Evol. Biol. **40**, 57–75 (2013).

[80] I. L. Dryden, K. Mardia, Statistical Shape Analysis (John Wiley & Sons, 1998).

[81] D. C. Adams, E. Otarola-Castillo, Geomorph: An R package for the collection and analysis of geometric morphometric shape data. Methods Ecol. Evol. **4**, 393–399 (2013).

[82] S. Schlager, “Morpho and Rvcg—Shape Analysis in R” in Statistical Shape and Deformation Analysis, G. Zheng, S. Li, G. Szekely, Eds. (Academic Press, 2017), pp. 217–256.

