# Supplementary material for: Insights into Neanderthal development, childbirth, and locomotion from the Palomas and Dederiyeh pelves
Source: Proc Natl Acad Sci U S A. 2026 Aug 17;123(35):e2535303123. doi: 10.1073/pnas.2535303123 (PMC13535174; doi:10.1073/pnas.2535303123)
Supplement: Supplementary file 1 [file pnas.2535303123.sapp.pdf]

PNAS

Supporting Information for

New insights into Neanderthal development, childbirth, and locomotion from the Palomas and Dederiyeh pelves

Authors:

Christoph P. E. Zollikofer[a,b]

Marcia S. Ponce de León[a,b]

María Haber Uriarte[c]

Mariano V. López[d]

Jon Ortega[d]

Osamu Kondo[e]

Takeru Akazawa[f]

Michael J. Walker[d]

[a] Department of Informatics, University of Zurich, 8050 Zurich, Switzerland

[b] Center for Climate Physics, Institute for Basic Science, Busan 46241, Republic of Korea

[c] Department of Prehistory, Archaeology, Ancient History, Mediaeval History and Historiographical Studies, Faculty of Letters, University of Murcia, Campus de La Merced, 30001 Murcia, Spain

[d] Murcian Association for the Study of Palaeoanthropology and the Quaternary, c/o Museo Arqueológico de Murcia, 30008 Murcia, Spain

[e] Department of Biological Sciences, Graduate School of Science, University of Tokyo, Tokyo 113-0033, Japan

[f] Research Institute, Kochi University of Technology, Kochi 782-8502, Japan

Corresponding Authors: zolli@ifi.uzh.ch, marcia@ifi.uzh.ch, mjwalke@gmail.com

This PDF file includes:

Supporting text
Figures S1 to S12
Tables S1 to S6
Legends for Datasets S1 to S2
SI References

Other supporting materials for this manuscript include the following:

Datasets S1 to S2

Table of Contents

1. Virtual reconstruction of the Dederiyeh 1 Neanderthal infant pelvis 3
2. Radiometric dating and the Neanderthal skeleton SP96 (“Paloma”) 4
3. Reconstruction of the “Paloma” Neanderthal female pelvis 4
4. Virtual reconstruction of the Tabun and Kebara adult Neanderthal pelves 8
5. Samples, landmarks, geometric morphometric analyses 8
6. Comparative analysis of infant pelvic shape variation 13
7. Comparative analysis of adult pelvic shape variation 14
8. Comparison of adult human and Neanderthal modes of pelvic sexual dimorphism 15
9. Comparative morphology of obstetrical planes 16
10. Acetabular orientation and pelvic orientation 17
11. Pelvic shape and locomotor biomechanics 19
12. Pelvic shape and birth mechanisms 21
13. Obstetric and locomotor constraints 24
14. Pelvic size, shape and the thermoregulation hypothesis 24
Dataset S1 Caption. 26
Dataset S2 Caption. 26
SI References 27

Figures

Fig. S1. Virtual reconstruction of the Dederiyeh 1 Neanderthal infant pelvis. 3
Fig. S2. Virtual reconstruction of the in-situ state of the “Paloma” pelvis SP-96. 5
Fig. S3. Virtual reconstruction of the “Paloma” pelvis SP-96. 7
Fig. S4. Anatomical landmarks visualized on adult pelvic mean shape. 12
Fig. S5. Morphometric analysis of the Dederiyeh 1 Neanderthal infant pelvis. 13
Fig. S6. PCA of pelvic shape variation in adult humans and Neanderthals 14
Fig. S7. Pelvic sexual dimorphism in adult Neanderthals and humans. 15
Fig. S8. Inlet, midplane and outlet dimensions. 16
Fig. S9. Pelvic reference planes and acetabular orientation. 18
Fig. S10. Femoropelvic biomechanical measurements. 20
Fig. S11. Pelvic dimensions and their relationship with FHC distance 23
Fig. S12. Pelvic surface-to-volume ratios in humans and Neanderthals. 25

Tables

Table S1. Sample structure 9
Table S2. Landmarks 10
Table S3. Measurements 12
Table S4. Correlations between modes of shape variation 14
Table S5. Femoropelvic measurements 20
Table S6. Statistical reevaluation of pelvic typology 22

1. Virtual reconstruction of the Dederiyeh 1 Neanderthal infant pelvis

The virtual reconstruction of the almost completely preserved pelvis of the ~1.5 year-old Neanderthal infant from Dederiyeh Cave (1) was based on a total skeletal reconstruction performed earlier (2). Since all pelvic elements are still unfused, a modern human sample of similar-aged infants (anonymized retrospective CT scan data, see Table S1A) was used to estimate the dimensions of the soft-tissue regions between the developing bony elements (Fig. S1). The position and orientation of the bony elements relative to each other, however, were inferred from local anatomical constraints, such as matching auricular regions of the sacral and iliac elements, the form of the acetabular cavity, and general bilateral symmetry.

Using modern human soft tissue data as a guide is likely to bias the Dederyieh pelvic reconstruction towards a "human-like" morphology. Nevertheless, this approach yields conservative estimates of the differences in pelvic morphology between Neanderthal and human infants.

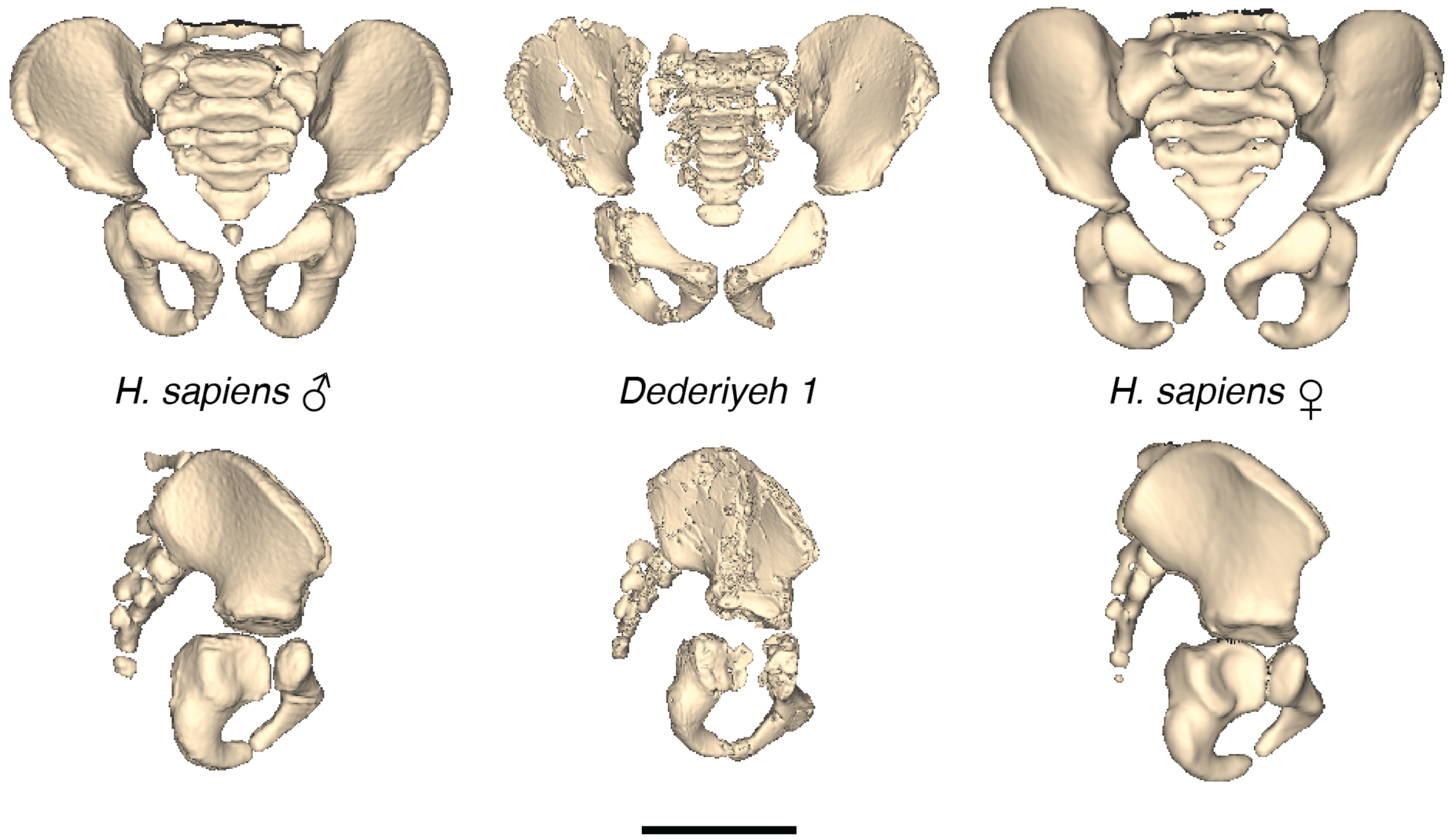

Figure S1. Virtual reconstruction of the Dederiyeh 1 Neanderthal infant pelvis. The reconstructed fossil pelvis (estimated individual age 1.5 years) is shown between male (left) and female (right) modern human infant pelves that are similar in size and developmental stage to Dederiyeh, but slightly older (~2 years). The scale bar is 5 cm.

2. Radiometric dating and the Neanderthal skeleton SP96 (“Paloma”)

The female skeleton SP96 (“Paloma”), to which the pelvis belongs (3–5), provided the ^{230}Th-^{234}U determination on a metacarpal bone of APSLP-1 of 54.1±7.7 ka (6) with 95% confidence interval CI of 61.6-46.6 ka. An animal bone fragment provided APSLP-6 of 51.0±2.5 ka, 95% CI 53.5-48.5 ka; it was excavated in a similar depth to where the Neanderthal skeletons SP96 (“Paloma”), SP97 (child) and SP92 (adult) lay in the porous, incompletely-cemented rock-tumble (“conglomerate A”; sedimentary Phase 2) that formed a sloping brecciated talus alongside which the fragment was excavated in sediments (Phase 3) that had accumulated around it. Greenland ice-core (GI) palaeotemperature records indicate “predominantly mild” (7) climatic interludes within Marine Isotope Stage MIS-4 between periods of intense cold during the ten millennia before ~40 ka. Thermophilous and mesophilous trees grew near Cabezo Gordo during one such interlude, because analysis of pollen (8, 9) in samples of the Phase 3 sedimentary accumulation banked up alongside the (Phase 2) “conglomerate A”, shows that some trees identified succumbed to frost, and therefore lived *before* the subsequent development of the intensely cold conditions prevalent in Western Europe ~39.9 ka. From 0.15-0.2 m above APSLP-6, another animal bone fragment, in a lens of burnt sediment, gave APSLP-4 43.8±1.5 ka, 95% CI 45.3-42.3 ka (6). Optical stimulated luminescence (OSL) dating of quartz grains (6) from sediment sample X2509 extracted from that lens gave 54.7±4.7 ka cal BP; its 1σ standard error allows a 95% CI of ~64-~45 ka. Below SP96, SP97 and SP92, lay the impermeable, strongly cemented “conglomerate B” layer that yielded U-series determinations of 67,700±4050 and 65,070±3800y BP (10) and 56,000 +13,000/-10,000y BP (11), corresponding to an intensely cold period ~70~62 ka within MIS-4 identified by GI researchers. Below “conglomerate B”, sedimentary Phase 1 has given U-series determinations of 90,300±7,300y BP (X6893;V5), 97,000±9,000y BP (X6890;V2), 102,100±12,000y BP (X6889;V1), 125,000±13,600y BP (X6891;V3), and 130,200±11,900y BP (X6892;V4) indicating MIS-5 (10, 12). Research on OSL is ongoing at the University of Oxford. In May 2026, Professor Jean-Luc Schwenninger and Marie Curie Fellow Mariana Sontag-González returned to the site to collect new samples from the sedimentary sequence.

3. Reconstruction of the “Paloma” Neanderthal female pelvis

Recovery and in-situ state. SP-96 (“Paloma”) was excavated in 2006-2007 from conglomerate **A** of the Late-Pleistocene deposits of Sima de las Palomas del Cabezo Gordo, Torre Pacheco, Murcia, Spain (3). All skeletal elements were preserved in anatomical connection but partially fragmented and distorted by taphonomic forces. The skeleton lay extended, on its right side, in an inclined position such that the head was at a slightly higher level than the legs. Both arms were folded upwards at the elbows, such that the hands were together beside the skull. Lying transversely in front of the right femur, the left femur was found as though drawn upwards. The pelvis was recovered in several blocks, taking account of the contact points between the blocks for subsequent reconstruction of its in-situ state (Fig. S2). The pelvis collapsed in the anteroposterior (dorsoventral) direction during fossilization. As an effect, several regions underwent in-situ fracturing and displacement. Displacement mainly occurred in anteroposterior and superoinferior directions. In transverse direction, the pelvic elements were held in position by the above-mentioned peculiar arrangement of the legs: both femoral heads were still in their respective acetabula, but the left femur was strongly flexed medially, crossing the right femur and fixated to it by dense sediment. The degree of femoral flexion, which is clearly beyond the normal physiological range of movement, indicates perimortem modification – be it in the form of funerary practices, a fatal fall, or early postmortem sedimentation.

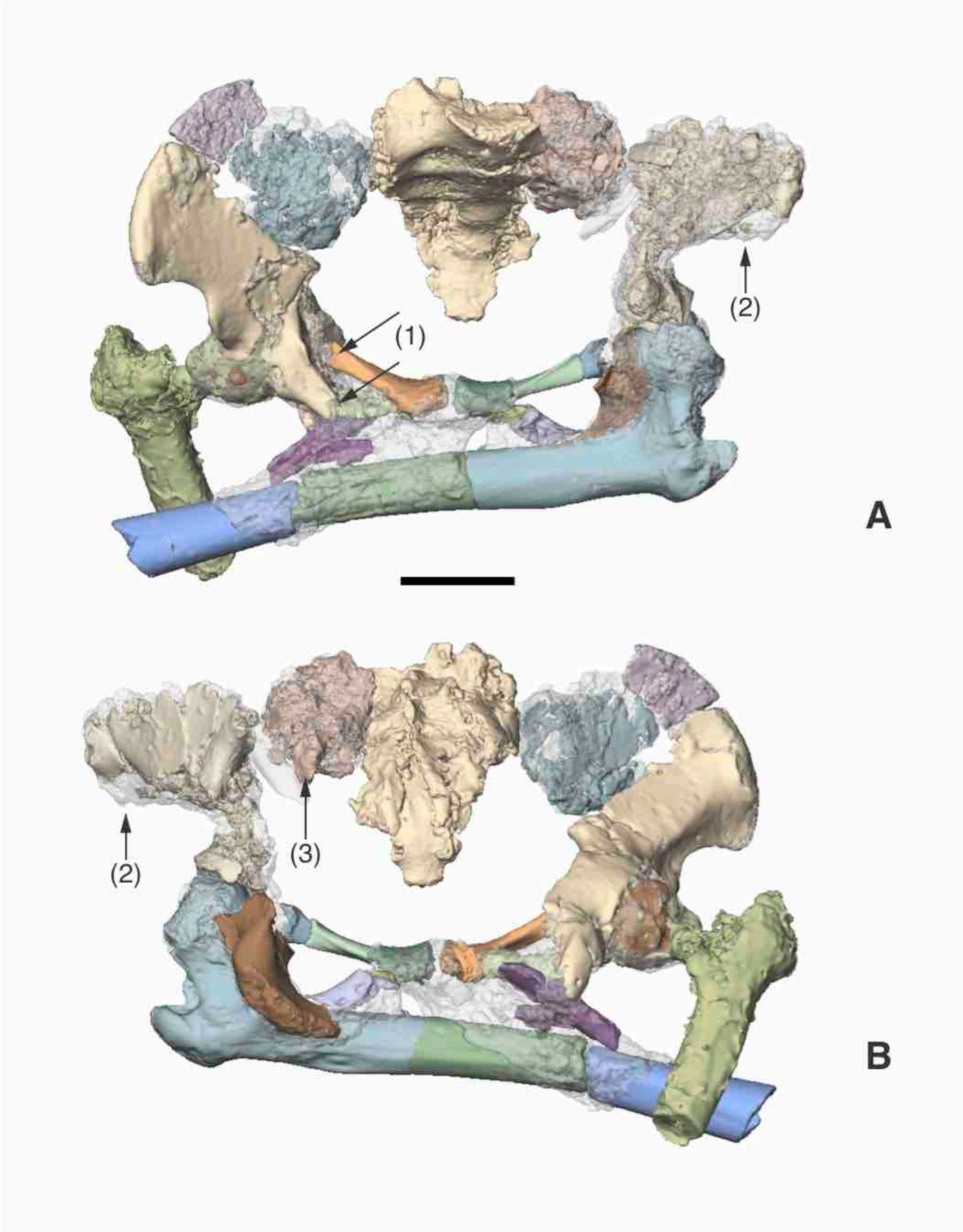

Figure S2. Virtual reconstruction of the in-situ state of the "Paloma" pelvis SP-96. (*A*) Anterosuperior view. (*B*) Posteroinferior view. Arrows: (1) postmortem fracture and anteroposterior displacement of right pubic ramus; (2) distorted left iliac blade; (3) left posterior inferior iliac spine in anatomical association with left sacrum fragment. Transparent parts denote digitally removed matrix.

Lab cleaning. Calcium-carbonate matrix adhering to exposed bone was removed using a CTS vibroincisor (C.T.S. España S.L.). Where bone was not exposed, calcium-carbonate conglomerate was dissolved using dilute acetic acid but as soon as a bone surface was exposed the conglomerate block was washed under running water for at least 24 hours and the exposed bone surface was then impregnated with Paraloid resin (Rohm & Haas Co., Philadelphia); after which, further removal commenced of surrounding calcium-carbonate using the vibroincisor, with further exposure of bony areas being impregnated with resin. Mechanical removal was used as little and as gingerly as possible, and only for removing stones cemented to bone surfaces by calcium carbonate that had resisted dissolution by acid.

CT scanning. At an early stage of preparation, a first set of medical CT data was acquired at the Veterinary Clinic of the University of Murcia and was used to guide further physical preparation work. Matrix regions harder than bone were left unprepared to prevent potential damage to the encased fossil parts. Subsequently, a second set of CT data was acquired at a higher resolution at the Clínica Quirón in Murcia. These data served as a basis for further digital preparation of the SP-96 pelvic remains. Due to the high variability in the X-ray densities of the matrix and bone, virtual preparation was performed using Slicer 3D's open-source interactive segmentation tools (13).

Recovering the in-situ position of the pelvic fragments. Combining the two-stage CT scan data with detailed records of the contact regions between the isolated original fossil parts allowed the in-situ position and orientation of the SP-96 pelvic remains to be re-established (Fig. S2). In its present physical state, the pelvis consists of several isolated fragments. Both pubic bones are represented by their rami and parts of their bodies and acetabular portions. The proximal and distal parts of the right ramus are displaced relative to each other in-situ. The left ramus disintegrated into three parts during recovery, but without further damage to the fracture sites, which permitted seamless re-assembly. Mirror-matching of left and corresponding right pubic fragments shows that this region did not undergo post-mortem distortion.

The ischial portion of the obturator foramen on the right side could be recovered by virtual preparation and repositioning of its constituent fragments. These were mirror-matched with the corresponding regions preserved on the left side.

The largest fragment from the right side comprises the well-preserved anterior part of the iliac blade, the acetabulum and parts of the ischium, all in anatomical connection. Mirror-matching with the well-preserved but more fragmentary regions on the left side permitted reconstruction of the anterior parts of the ossa coxae. The posterior region of the right iliac blade is less well preserved and partially encased in extremely hard, grainy matrix. Importantly, however, this fragment exhibits anatomical connection with the sacrum. The sacrum is undistorted and well-preserved overall, except for the left auricular side, which is partially crushed. This side has no preserved anatomical connection to the left auricular surface and posterior iliac blade.

Virtual reconstruction of the pelvis. The inferred in-situ setting of the SP-96 pelvic fragments (Fig. S2) served as a starting point to correct for the effects of taphonomic fragmentation and displacement, and place all elements in standard anatomical space. Overall, the anatomical relationships between the sacral and coxal elements of the pelvic girdle are constrained by their anatomical relationships and by bilateral symmetry. The following constraints were observed:

a) the pelvic midplane is represented posteriorly by the sacrum, and anteriorly by the left and right pubic bodies forming the pubic symphysis;
b) the right side of the pelvic girdle is constrained by preserved anatomical connections from the sacrum to the pubic ramus;

c) the position and orientation of the fragmentary left iliac portions can be defined by mirror-imaging the better-preserved right counterpart across the well-established sacral midplane;

d) a small fragment of the left posterior inferior iliac spine adheres in anatomical connection with the left sacral auricular rim, and defines the superoinferior location of the sacrum relative to the iliac blades;

e) the anterior mediolateral width of the pelvis is constrained by the pubic symphyseal region, with some uncertainty arising from the variable breadth of the symphysis;

f) the biacetabular width is constrained by the two femora, which were preserved in crossed position and in a single matrix block, while their heads remained cemented in the acetabula.

The result of the virtual reconstruction is visualized in Fig. S3. Three independent virtual reconstructions were performed by MPdL and ChZo. These served as an estimate of "reconstructive uncertainty".

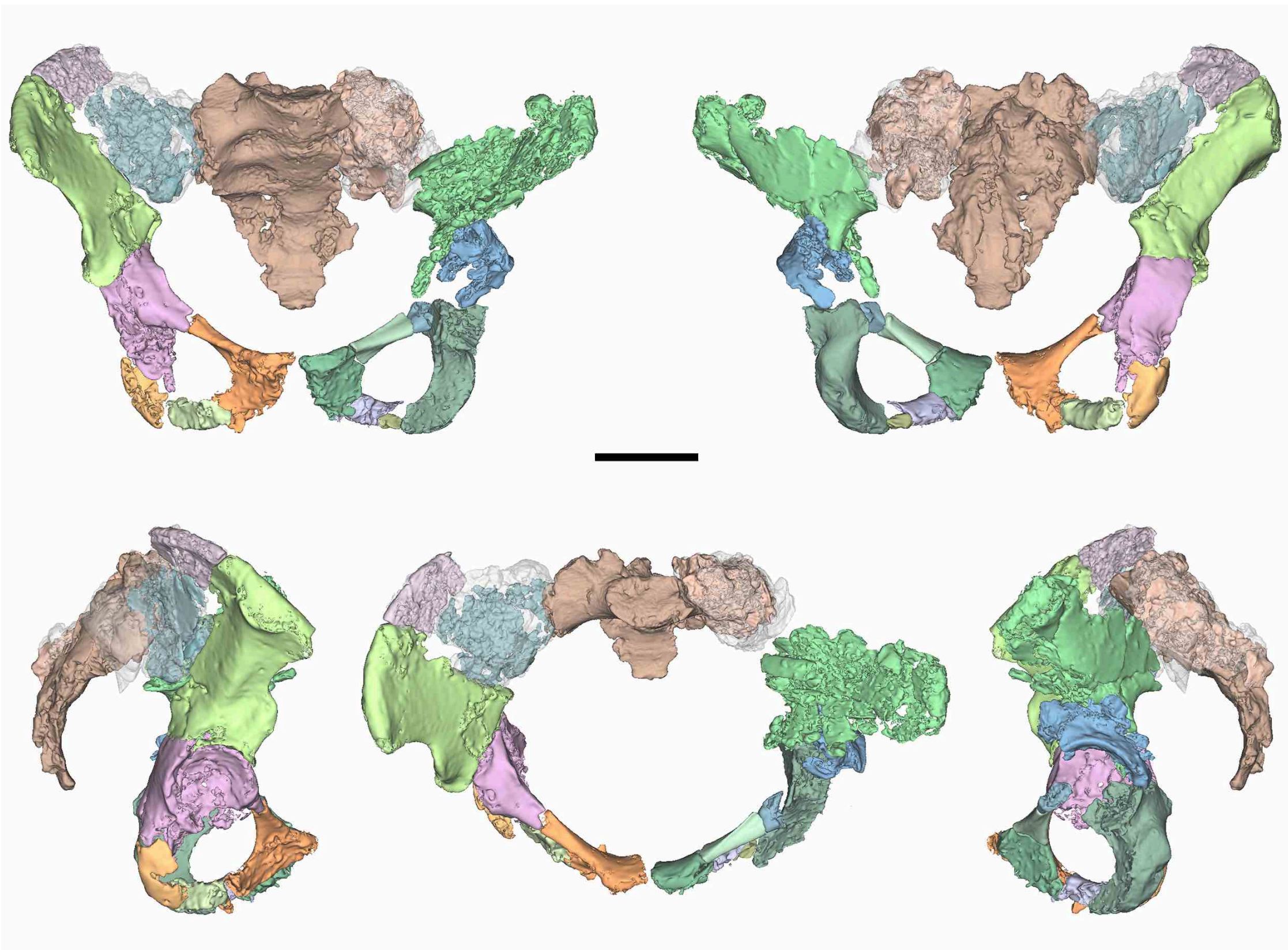

Figure S3. Virtual reconstruction of the "Paloma" pelvis SP-96. From left to right and top to bottom: Anterior, posterior, right lateral, anterosuperior and left lateral views. The iliac crests as well as the sacral bodies are only partially fused, suggesting an age at death between 15 and 20 years according to modern human standards.

4. Virtual reconstruction of the Tabun and Kebara adult Neanderthal pelves

The Tabun female and Kebara male pelves were first described in detail in refs. (14) and (15), respectively. We re-used the virtual reconstructions performed earlier (2). The missing sacroiliac region of Tabun was inferred using the corresponding regions of the Palomas and the Kebara pelves, and applying Thin-Plate Spline morphing based on anatomical landmarks (see below). The cranial dimensions of the neonate Neanderthal from Mezmaiskaya Cave served as an additional constraint for transverse and anteroposterior maternal pelvic dimensions, assuming that Tabun must have been able to give birth to a neonate with the Mezmaiskaya cranial dimensions (2). An alternative reconstructive approach (16) used multiple regression and expectation-maximization on a modern human comparative sample to estimate the missing parts of the Tabun pelvis. However, this study yielded obstetrical dimensions that were too small for successful delivery (17). This bias is likely an effect of the pronounced modularity of the hominin pelvis (18): Multivariate regression uses the human-specific pattern of covariance between pelvic elements as the basis for the Neanderthal pelvis; however, Neanderthal pelvic form likely exhibited a different pattern of modularity.

5. Samples, landmarks, geometric morphometric analyses

The pelvis develops from childhood to adulthood along a nonlinear trajectory (19). To manage the additional complexity of Neanderthal-human comparisons, we performed two independent morphometric analyses: one for the Dederiyeh 1 Neanderthal infant with an infant human comparative sample, the other one for the adult Neanderthals together with an adult human comparative sample. The respective sample structures are summarized in Table S1. Pelvic morphology was quantified with sets of three-dimensional anatomical points of reference (so-called landmarks); these are specified in Table S2.

Infant pelvic sample and anatomical landmarks

The virtual reconstruction of the Dederiyeh 1 Neanderthal infant pelvis was analyzed together with a comparative human infant sample of N_i=34 individuals aged 1 month to 8.4 years [anonymized retrospective CT scan data, as specified in ref. (19); see Table S1A]. The 3D anatomical landmarks used to quantify infant pelvic morphologies are listed in Table S2A. This landmark set differs from the adult set in the way how corresponding points on fusing pelvic elements are treated: In the infant pelves (pre-fusion), these points are represented by separate landmarks (Table S2A) while in adults (post-fusion) they are represented by a single landmark (Table S2B; Fig. S4).

Adult pelvic sample and anatomical landmarks

The adult Neanderthal sample comprises the Palomas, Tabun and Kebara pelves, each represented by three reconstructive variants. This sample was complemented by the Kebara 2 reconstruction of Adegboyega et al. (20), which is represented by three variants of 3D landmark data sampling. Landmarks in regions not preserved in a given fossil specimen were inferred with methods of thin-plate spline (TPS) interpolation, using morphologically similar specimens as templates. For example, landmarks in the missing ilio-sacral region of the Tabun specimen were inferred using the more complete Palomas and Kebara specimens as templates. We refrained from using population-based methods of inference, such as multivariate regression and expectation-maximization (16), as the highly modular organization of the hominin pelvis (18) limits inferences about missing Neanderthal pelvic regions based on a modern human sample.

The adult human comparative sample consists of retrospective clinical CT data of N_a=112 individuals aged 14-49 years, obtained from several sources, as specified in ref. (19). See Table S1B for a summary of the sample structure. The 3D anatomical landmarks used to quantify adult pelvic morphologies are listed in Table S2B and visualized in Fig. S4. We evaluated various linear and angular measurements from the original 3D landmark data in order to characterize pelvic dimensions relevant to obstetrics and biomechanics (see Table S3).

Geometric morphometric analyses
Geometric morphometric (GM) analyses were performed independently on the infant and adult samples, respectively, but using the same standard procedures (21) implemented in the R packages *geomorph* (22) and *Morpho* (23). Because natural patterns of pelvic bilateral asymmetry cannot be reliably recovered from fossil specimens, all analyses were performed on symmetrized specimens. Symmetrization was performed by averaging the original and mirror-imaged landmark configurations of each specimen. Generalized Procrustes Analysis (GPA) was used to size-standardize the specimens (centroid size =1) and iteratively align them through translation and rotation about their centroids to the sample-mean landmark configuration (the consensus configuration). Principal components analysis (PCA) of shape was then used to visualize the major patterns of shape variation in the sample in a low-dimensional subspace of multivariate shape space. Principal components (PCs) are statistically orthogonal axes that account for largest, second-largest etc. proportions of total variation. While PCs are well suited to exploring shape variation, they do not necessarily represent biologically relevant modes of variation. Note that size was not included as a variable in the PCAs, but later used as an external covariate of shape to evaluate allometric effects.

Table S1A. Sample structure: infants

H. sapiens	*N*	*age*
females	15	1 month – 8.5 years
males	19	1 month – 8.5 years
H. neanderthalensis		*estimated age*
Dederiyeh 1	1	1.5 years

Table S1B. Sample structure: adults

H. sapiens	*N*	*age (y)*
females	56	14 – 49
males	56	14 – 46
H. neanderthalensis		*estimated age (y)*
Palomas female	3 reconstruction variants	15 – 20
Tabun female	3 reconstruction variants	20 – 40
Kebara male, this study	3 reconstruction variants	20 – 40
Kebara male, ref. (20)	3 landmark data acquisition variants	

Table S2A. Anatomical landmarks of infant pelves.

landmarks (right – mid – left)			*description*
fixed landmarks			
1		2	pubic symphysis: superiormost anterior point
3		4	pubic symphysis: superiormost posterior point
5		6	ischiopubic juncture: pubis - posterior fusion point
7		8	ischiopubic juncture: ischium - posterior fusion point
9		10	ischiopubic juncture: pubis - obturator foramen inferior fusion point
11		12	ischiopubic juncture: ischium - obturator foramen inferior fusion point
13		14	ischiopubic juncture: pubis - obturator foramen superior fusion point
15		16	ischiopubic juncture: ischium - obturator foramen superior fusion point
17		18	ischium: posteriormost point (superior from ischial tuberosity)
19		20	ischium: inferiormost midpoint
21		22	ilioischial juncture: ischium - fusion point
23		24	ilioischial juncture: ilium - fusion point
25		26	ilioischial juncture: ischium - acetabulum lateral fusion point
27		28	ilioischial juncture: ilium - acetabulum lateral fusion point
29		30	acetabulum: superiormost lateral point
31		32	acetabulum: point on pubic part of lunate surface
33		34	iliopubic juncture: pubis - superior fusion point
35		36	iliopubic juncture: ilium - superior fusion point
37		38	iliopubic juncture: pubis - fusion point on pelvic brim
39		40	iliopubic juncture: ilium - fusion point on pelvic brim
41		42	pubis: anterior midpoint
43		44	acetabulum: inferiormost lateral point on lunate surface
45		46	acetabulum: deepest point on acetabular center (acetabular fossa)
47		48	pelvic brim + sacroiliac joint: intersection point
49		50	sacroiliac joint: superiormost point
	51		S1: superiormost anterior point
	52		S1: superiormost posterior point
	53		S1: anterior midpoint
	54		S2: anterior midpoint
	55		S3: anterior midpoint
	56		S4: anterior midpoint
57		58	ant. sup. iliac spine*
59		60	iliac crest: posteriormost point (end point)
61		62	post. inf. iliac spine*
	63		S5: anterior midpoint
64		65	pubis posterior
semilandmarks			
66 - 72		73-79	ischium posterior
80-82		83-85	obturator foramen anterior
86-88		89-91	obturator foramen posterior
92-94		95-97	acetabulum: on ischium
98-100		101-103	acetabulum: on ilium
104-106		107-109	pelvic brim1
110-112		113-115	pelvic brim2
116-118		119-121	sacroiliac joint
122-124		132-134	anterior iliac spines
125-131		135-141	Iliac crest
142-144		145-147	posterior iliac spines
148-150		151-153	greater sciatic notch

Table S2B. Anatomical landmarks of adult pelves.

landmarks (right – mid – left)			*description*
fixed landmarks			
1		2	pubic symphysis: superiormost anterior point
3		4	pubic symphysis: superiormost posterior point
5		6	ischiopubic juncture: pubis - posterior fusion point
7		8	ischiopubic juncture: pubis - obturator foramen inferior fusion point
9		10	ischiopubic juncture: pubis - obturator foramen superior fusion point
11		12	ischium: posteriormost point (superior from ischial tuberosity)
13		14	ischium: inferiormost midpoint
15		16	ilioischial juncture: ischium - fusion point
17		18	ilioischial juncture: ischium - acetabulum lateral fusion point
19		20	acetabulum: superiormost lateral point
21		22	acetabulum: point on pubic part of lunate surface
23		24	iliopubic juncture: pubis - superior fusion point
25		26	iliopubic juncture: pubis - fusion point on pelvic brim
27		28	pubis: anterior midpoint
29		30	acetabulum: inferiormost lateral point on lunate surface
31		32	acetabulum: deepest point on acetabular center (acetabular fossa)
33		34	pelvic brim + sacroiliac joint: intersection point
35		36	sacroiliac joint: superiormost point
	37		S1: superiormost anterior midpoint
	38		S1: superiormost posterior midpoint
	39		S1: anterior midpoint
	40		S2: anterior midpoint
	41		S3: anterior midpoint
	42		S4: anterior midpoint
	45		S5: anterior midpoint
	46		pubic symphysis ventralmost midpoint
	47		sacro-coccygeal joint
43		44	iliac crest: posteriormost point
48		49	iliac tubercle lateralmost point
50		51	anterior superior iliac spine
52		53	inferior gluteal line (ant. inf. iliac incisure)
54		55	anterior inferior iliac spine
56		57	posterior superior iliac spine, most posterior point
58		59	posterior inferior iliac spine, anterior point
60		61	posterior inferior iliac spine, posterior point
62		63	ischial spine
64		65	incisura ischiadica
66		67	obturator foramen medialmost superior point
68		69	obturator foramen medialmost inferior point
70		71	obturator foramen superiormost lateral point
72		73	obturator foramen inferiormost lateral point
74		75	widest points on pelvic brim
76		77	pubis posterior
semilandmarks			
78-84		85-91	ischium posterior
92-94		95-97	pelvic brim1
98-100		101-103	pelvic brim2
104-110		111-117	iliac crest
118-120		121-123	greater sciatic notch

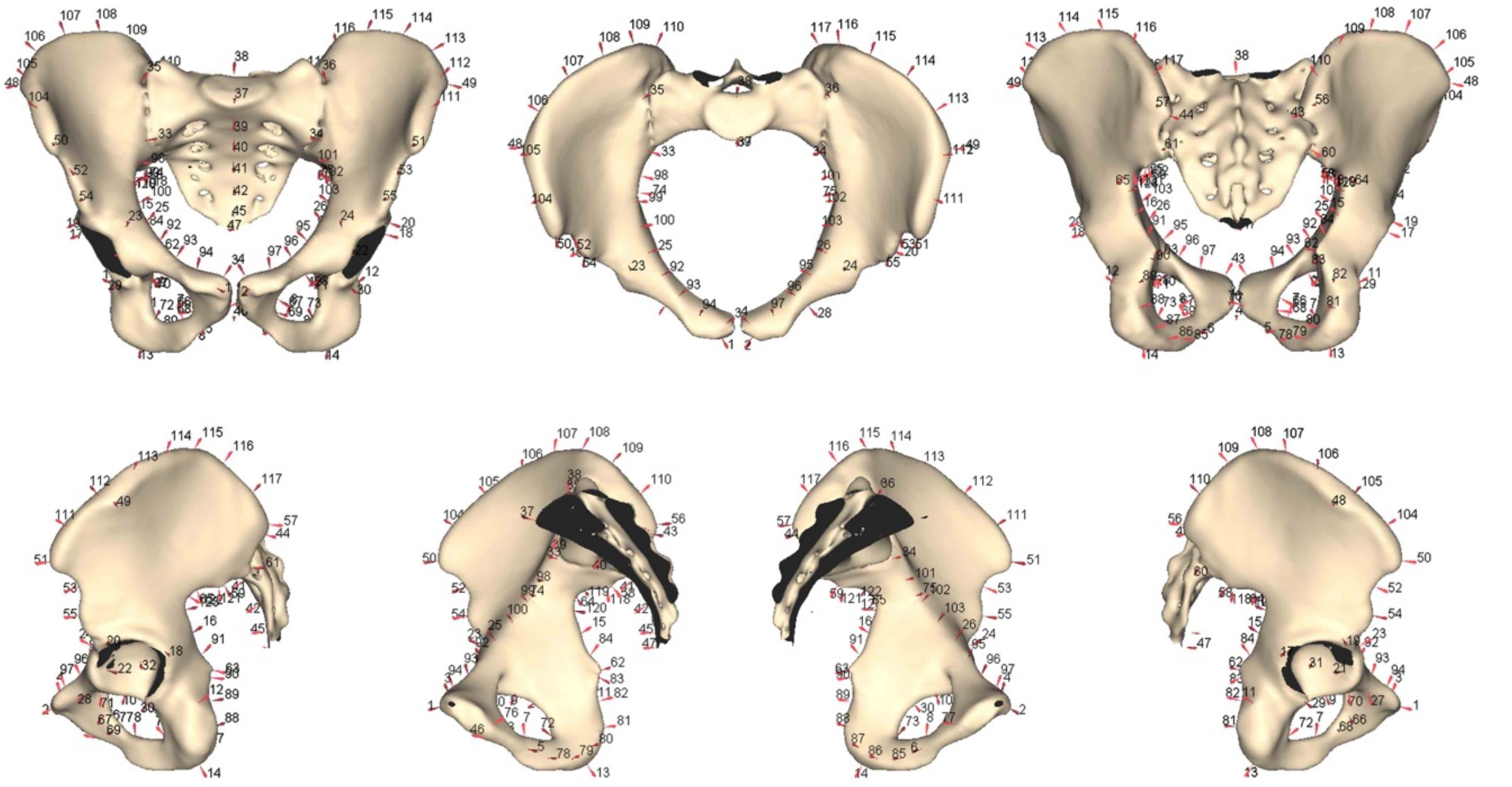

Figure S4. Anatomical landmarks visualized on adult pelvic mean shape (see Table S2B).

Table S3. Definition of measurements and anatomical planes of the adult pelvis.

linear measurements	*name*	*dimension*	*evaluation**
inlet transverse	ap_{in}	mm	dist [74, 75]
inlet anteroposterior	tr_{in}	mm	dist [37, mean(3,4)]
midplane transverse	ap_{mid}	mm	dist [62, 63]
midplane anteroposterior	tr_{mid}	mm	dist [mean(42,45), mean(76,77)]
outlet transverse	ap_{out}	mm	dist [80 87]
outlet anteroposterior	tr_{out}	mm	dist [47, mean(76,77)]
biiliac		mm	dist [48, 49]
distance between femoral head centers (FHC) = biacetabular central width	w_{FHC}	mm	left FHC to right FHC
sacroacetabular distance	d_{sa}	mm	dist [mean(31,32), mean(37,38)]
indices and areas			
inlet index	i_{in}	%	$ap_{\text{in}} / tr_{\text{in}} \cdot 100$
midplane index	i_{mid}	%	$ap_{\text{mid}} / tr_{\text{mid}} \cdot 100$
outlet index	i_{out}	%	$ap_{\text{out}} / tr_{\text{out}} \cdot 100$
inlet area	a_{in}	cm^2	$ap_{\text{in}} \cdot tr_{\text{in}} \cdot \pi / 400$
midplane area	a_{mid}	cm^2	$ap_{\text{mid}} \cdot tr_{\text{mid}} \cdot \pi / 400$ (**)
outlet area	a_{out}	cm^2	$ap_{\text{out}} \cdot tr_{\text{out}} \cdot \pi / 400$ (**)
planes, vectors and angles			
coronal pelvic plane	*CPP*		plane through acetabular centers (31, 32) and S1 anterior point (37)
sacroacetabular plane	*CPP'*		plane through acetabular centers (31, 32) and center of S1 surface [mean of (37, 38)]
transverse plane	*TPP*		plane through 31, 32, normal to *CPP*
acetabular orientation	*AO*		normal vector to plane through acetabular rim (17, 19, 29)
acetabular inclination	α	°	angle between *AO* and *TPP*
acetabular ante-/retroversion	θ	°	angle between *AO* and *CPP*

* numbers indicate landmark ids in Table S2A
** area S of ellipse with major and minor diameters a, b: $S = \pi\, a\, b / 4$

6. Comparative analysis of infant pelvic shape variation

We evaluated how pelvic shape changes as a function of individual age and increasing size, and how the pelvis of the Dederiyeh 1 Neanderthal differs from that of modern human infants, taking into account the effects of size and age. The results of these analyses are presented in main Figs. 2A and 3A, and in Fig. S5. Principal shape component PC1 captures age- and size-related changes in pelvic shape (Fig. S5A). The Dederiyeh 1 Neanderthal infant is at the upper end of human pelvic shape and size variation for its age of about 1.5 years (Fig. S5A). Higher-order PCs account for shape variation in the sample regardless of age (Fig. S5B). Dederiyeh 1 markedly deviates from the human sample along age-independent shape PC4. This mode of shape variation is visualized as follows (Fig. 3D, Fig. S5C): a human specimen representing a similar developmental status (inferior pubic rami almost reaching ischial ramus) but slightly older (2 years) than Dederiyeh 1 was morphed to the sample mean shape and visualized as the yellow specimen in Fig. S5C. The mean shape was then deformed according to the PC4 coordinate value of the Dederiyeh 1 specimen, and the resulting shape was superimposed (blue) on the mean shape.

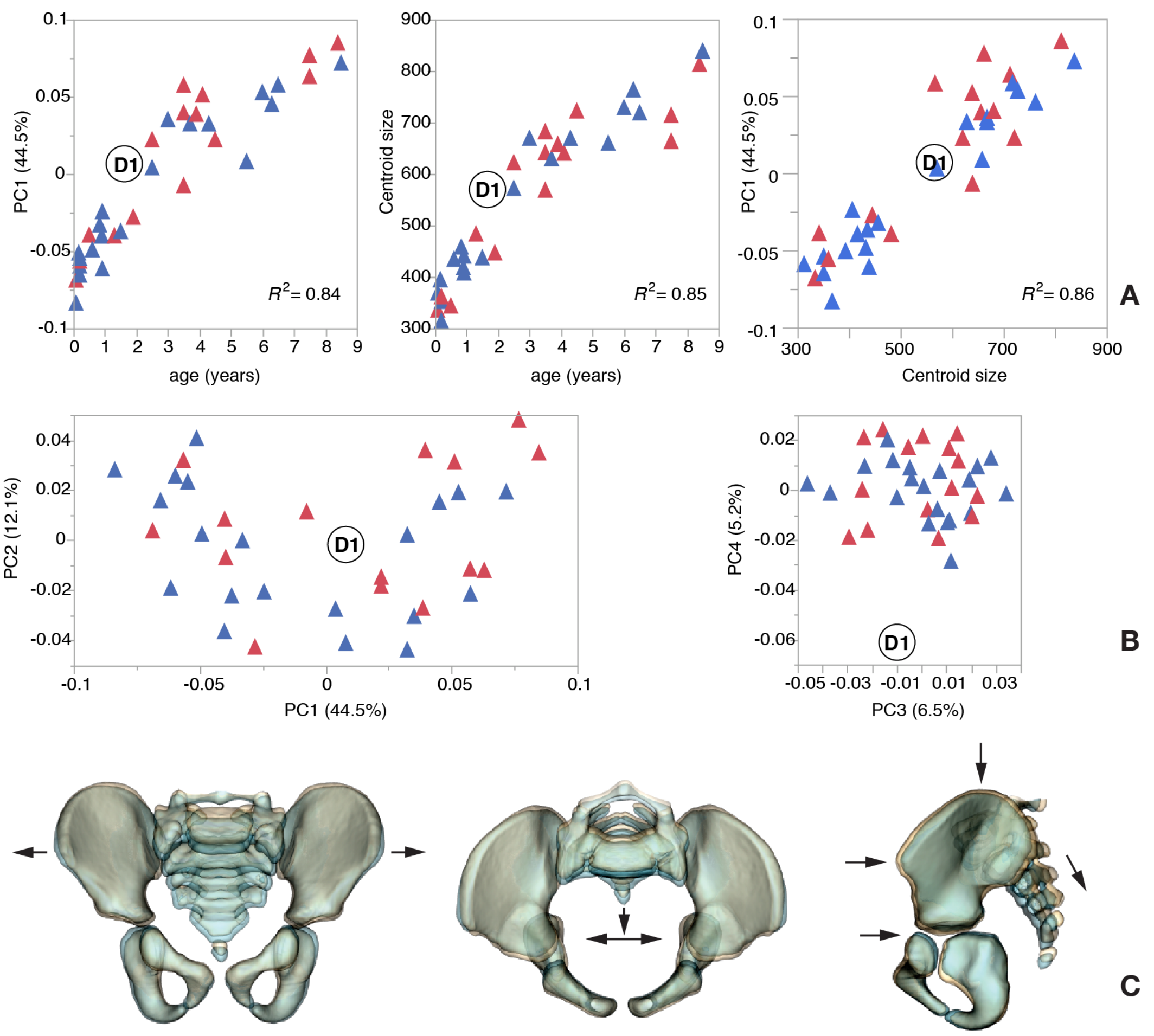

Figure S5. Morphometric analysis of the Dederiyeh 1 Neanderthal infant pelvis. Red/blue triangles: modern human female/male infants. D1: Dederiyeh 1 Neanderthal infant. (*A*) Shape component PC1 accounts for 44.5% of the total shape variation in the sample and is strongly correlated with age and pelvic centroid size. Dederiyeh 1 is at the upper end of modern human age- and size-related shape variation. (*B*) Location of Dederiyeh 1 in morphospace. Shape component PC4 separates D1 from modern human infants independent of age and size. (*C*) Modern human-to-Neanderthal infant mode of pelvic shape variation captured along PC4.

7. Comparative analysis of adult pelvic shape variation

We evaluated three biologically relevant modes of shape variation: variation between species (Neanderthals vs. humans), variation between sexes (females vs. males), and variation within species (only for the human sample: direction of the first PC of human pooled-sex variation). These modes (directions of shape variation) are visualized as colored arrows in Fig. 2B and Fig. S6. Each of these modes of shape variation can be represented by a vector through multivariate shape space. Evaluating the angles between these vectors informs about possible correlations between intra-specific, interspecific and sex-specific pelvic variation. Table S4 lists the angles between the mode vectors, and the corresponding R^2 values that indicate the proportion of shared variance. The small proportions of shared variance indicate fairly independent patterns of variation.

We further evaluated how adult pelvic shape varies as a function of size (static allometry). Multivariate regression of shape on log (centroid size) revealed only minor effects (R^2=0.02; see black arrows in Fig. 3B), which are not considered further in this study.

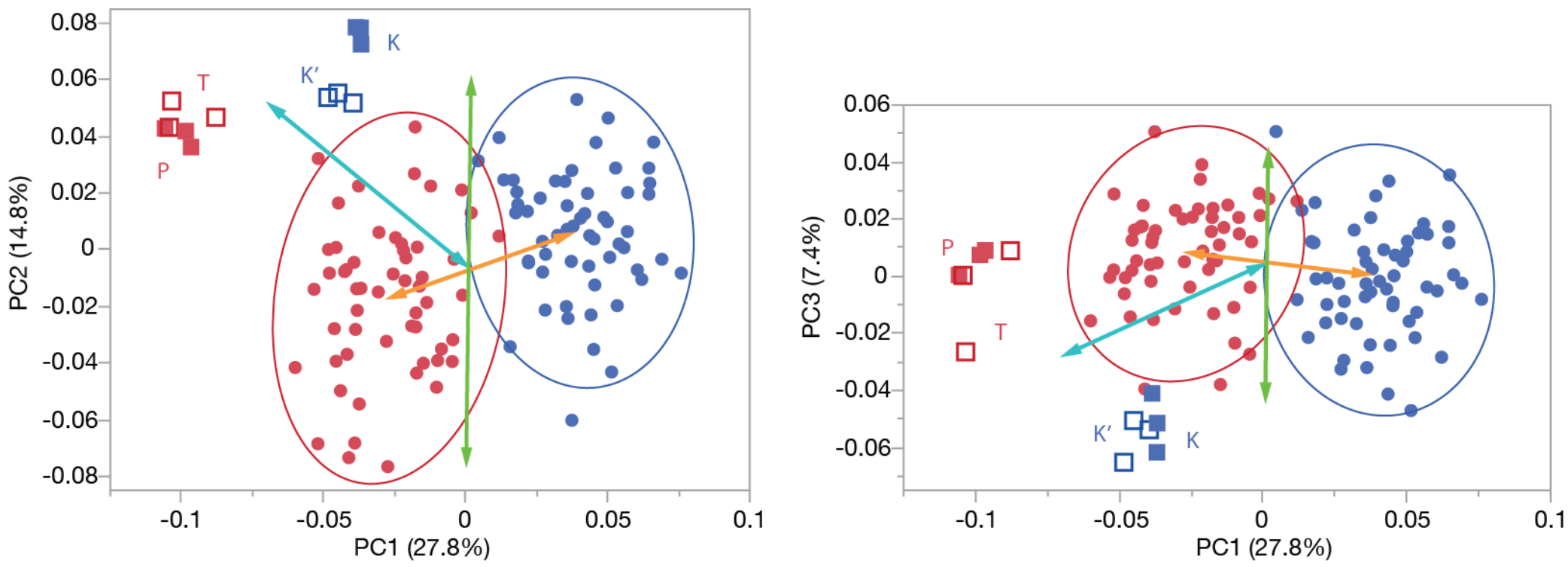

Figure S6. Principal components analysis of pelvic shape variation in adult humans and Neanderthals. Red: females; blue: males; circles: *H. sapiens* (95% ellipses around female and male distributions); squares: *H. neanderthalensis*; filled symbols: Palomas (P) and Kebara (K) fossil reconstructions; open symbols: Tabun (T) from ref. (2) and Kebara (K') from ref. (20). Each fossil specimen is represented by three reconstructive variants. Colored arrows indicate major modes of shape variation; cyan: Neanderthal mean ⟷ human mean, orange: human female mean ⟷ male mean; green: major mode of shape variation within humans, independent of sex (projected on human mean; length = 2 standard deviations).

Table S4. Correlations between major modes of adult pelvic shape variation.

	*intra-species** *vs.* *female-male*	*intra-species* *vs.* *Neanderthal-human*	*Neanderthal-human* *vs* *female-male*
angle between modes (°)	79.7	73.5	64.4
coefficient of determination (R^2)	*0.031*	*0.080*	*0.186*

* Colors correspond to the arrows shown in Fig. S6.

8. Comparison of adult human and Neanderthal modes of pelvic sexual dimorphism

The aim here is to establish whether pelvic sexual dimorphism (PSD) in Neanderthals is similar in pattern and magnitude to that of humans (Fig. S7). In our human sample, PSD is best represented by the vector in shape space that connects female and male mean shapes: $\mathbf{v}_{\mathrm{Hs.mean}}$. Since the Neanderthal sample only consists of two females and one male, its mean PSD vector $\mathbf{v}_{\mathrm{Hn.mean}}$ is unlikely to be a good estimate of the true Neanderthal mean PSD vector. We cannot therefore directly compare $\mathbf{v}_{\mathrm{Hs.mean}}$ with $\mathbf{v}_{\mathrm{Hn.mean}}$. As an alternative, we resample K_{Hs}=100 female-male (i,j) pairs from the adult *H. sapiens* sample (N=56 males and females, respectively; see Table S1B). For each of these pairs, a PSD vector $\mathbf{v}_{\mathrm{Hs}.ij}$ is computed in shape space (PCs1-3; higher-order PCs do not show significant differences between sexes). Similarly, *H. neanderthalensis* vectors $\mathbf{v}_{\mathrm{Hn}.kl}$ are computed between all possible adult female-male reconstruction variant pairs (k,l) (see Table S1A; K_{Hn}=36). These shape space vectors correspond to real-space differences between the respective female-male pairs in terms of pattern (vector direction) and magnitude (vector length). We then computed the angles ϕ between all possible vector pairs and evaluated the frequency distributions of ϕ for human-human, Neanderthal-Neanderthal and Neanderthal-human pairs. Fig. S7A shows that Neanderthal-human ϕ values are well within the distribution of human-human ϕ, indicating that the pattern of Neanderthal PSD is within the range of variation displayed by humans. Furthermore, we evaluated the frequency distribution of vector lengths for humans and Neanderthals. Fig. S7B indicates that the magnitude of Neanderthal PSD is within the human range of variation.

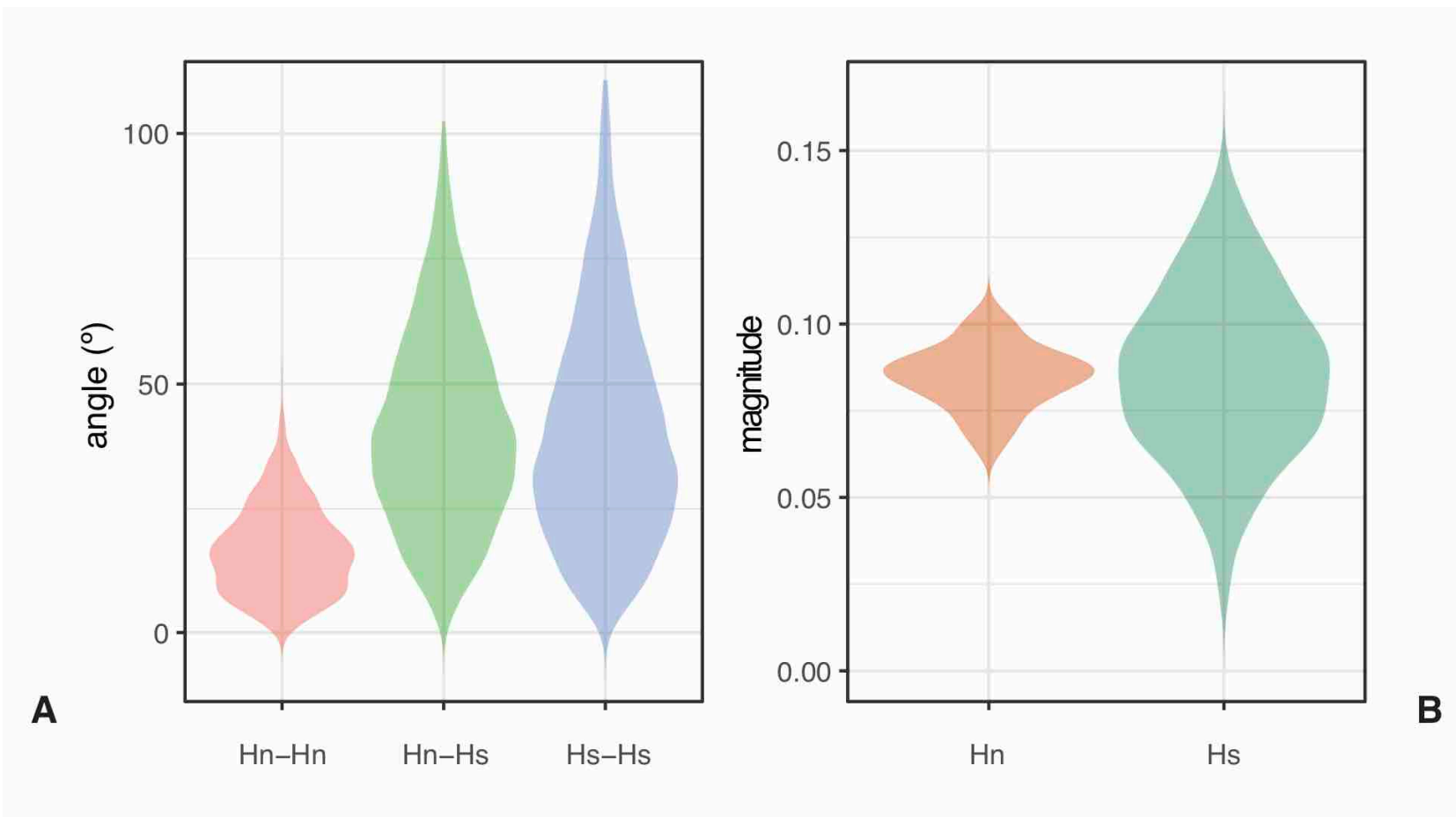

Figure S7. Pelvic sexual dimorphism (PSD) in adult Neanderthals and humans. (*A*) Frequency distribution of angles (measured in multidimensional shape space, see main Fig. 2 and Fig. S6) between all possible female-male (f-m) vectors of the Neanderthal sample (Hn-Hn), between all Neanderthal f-m vectors and 100 resampled human f-m vectors (Hn-Hs), and between 100 resampled human f-m vectors (Hs-Hs). These distributions show that there is substantial variation in the way in which the pelves of any female differs from that of any male (as an effect of substantial intraspecific variation independent of sex; see Fig. S6). Within Neanderthals (Hn-Hn), angular variation is comparatively small, due to the small effective Neanderthal sample size (reconstructive variants of two females and one male). (*B*) Magnitude (female-to-male vector length) of PSD for all possible Neanderthal female-male pairs (Hn) and for 100 resampled human female-male pairs (Hs).

9. Comparative morphology of obstetrical planes

The frequency distributions of sex-specific inlet, midplane and outlet areas and shape indices (see Table S3) are visualized in Fig. 4, complemented with data from individual fossil specimens representing *Australopithecus* and early *Homo* (24–27). Bivariate plots of the original data are presented in Fig. S8.

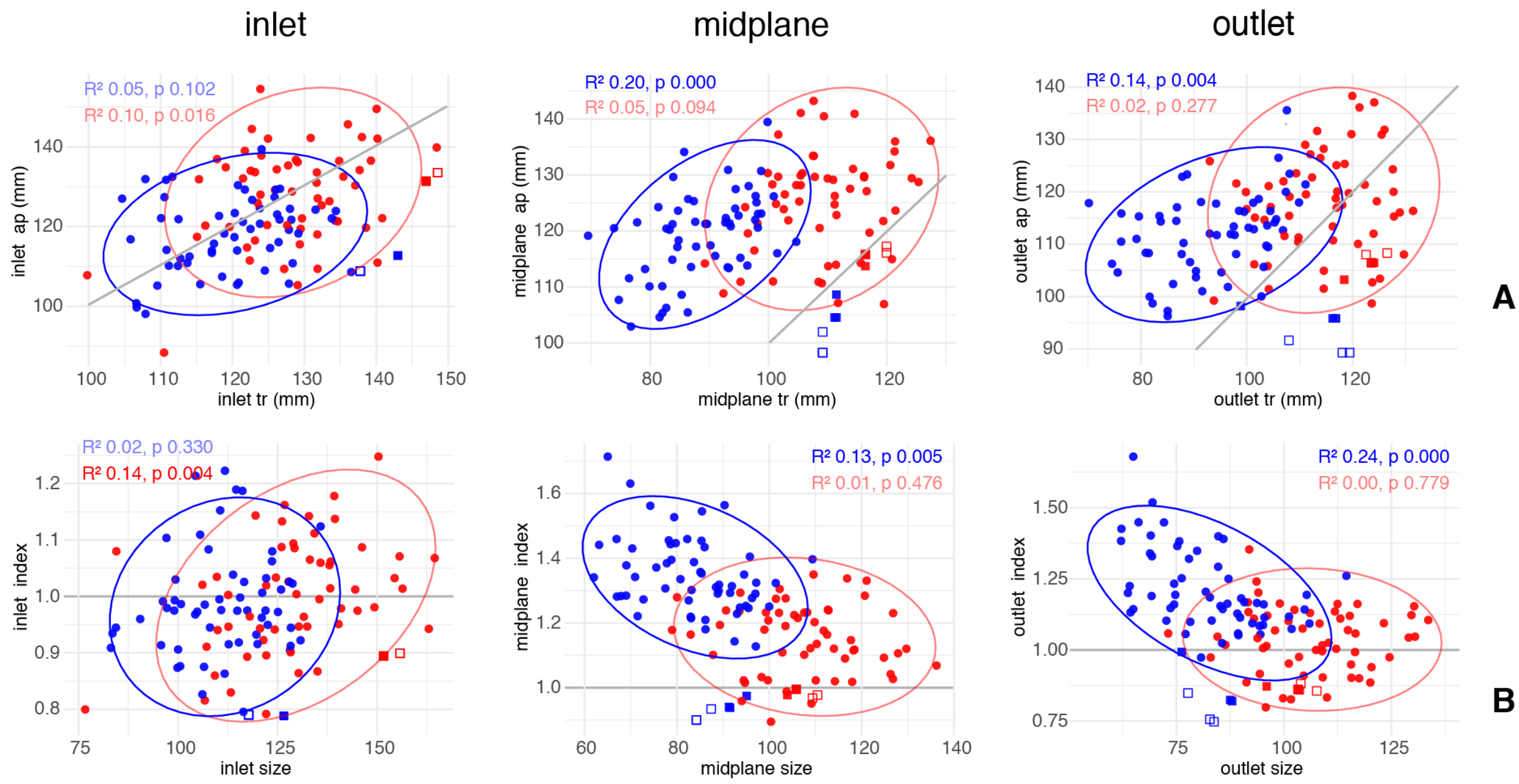

Figure S8. Inlet, midplane and outlet dimensions. Red/blue symbols: females/males. Circles: *H. sapiens*. Squares: *H. neanderthalensis*; filled: Palomas, Kebara; open: Tabun, Kebara (20) reconstructions. 95% ellipsoids, R^2 and p values for modern human females and males. Gray lines indicate indices=1. (*A*) Anteroposterior (ap) versus transverse (tr) dimensions. In human females, the midplane and outlet ap *versus* tr dimensions are not correlated; females tend to have larger tr dimensions than males. Neanderthal females have ap and tr dimensions at the low and high range of human female variation, respectively. The Kebara male Neanderthal falls outside the human range of ap-tr covariation, with larger tr but smaller ap dimensions. (*B*) Pelvic indices (ap/tr) versus area size (in cm^2). Human female midplane and outlet shape variation is independent of size variation. Overall, larger midplane and outlet sizes tend to be associated with more circular (less ap-elliptical) canal shapes, challenging the idea that ap-elliptical shapes have evolved to optimize pelvic floor stability (28).

10. Acetabular orientation and pelvic orientation

Drawing inferences on the biomechanics of locomotion from fossil hominin pelvic morphologies requires comparative data from living subjects, where morphological and actual locomotor properties can be studied simultaneously and compared with model simulations (29). The typical approach is to first characterize pelvic morphology in terms of intrinsic anatomical reference planes, then to study the pelvis' orientation in physical space, i.e., in relation to the direction of gravity (stance) and/or of ground reaction forces (locomotion). Here, we focus on those variables that are widely used in the literature to describe locomotion-related pelvic morphology. We critically examine which inferences can be drawn from intrinsic anatomical variables on the pelvis' spatial orientation.

Acetabular orientation (AO) is a parameter that is used in reconstructive hip surgery (30), as well as to infer a hominin fossil's biomechanics during stance and locomotion (31–33). AO is defined as the normal vector of the plane through the acetabular rim (see Fig. 5 and Table S3) (33). For interindividual comparisons, AO is typically expressed in terms of acetabular ante-/retroversion (θ) and inclination (α) relative to anatomical reference planes (34, 35). Here, two pelvic reference planes are defined to measure θ and α (Fig. S9A):

1) *Coronal pelvic plane (CPP)*: A transverse plane that runs through the right and left femoral head centers (FR, FL), and through the anterosuperior midpoint of the first sacral vertebra (S1). Acetabular ante-/retroversion θ is measured as the angle between the AO vector and the coronal plane. During stance, this plane is assumed to be approximately vertical (see Fig. 5, Fig. S9 and Table S3) and to be aligned with the center of mass of the trunk.
2) *Transverse plane*: A plane perpendicular to CPP, running through the FHCs. Acetabular inclination α is measured as the angle between AO and the transverse plane. This plane is assumed to be approximately horizontal during stance.

Fig. 5 (main manuscript) shows that, relative to CPP, the acetabula of human females are generally more anteverted than those of human males, which complements the evidence from clinical studies (34–37)]. With reference to CPP, the acetabula of the Neanderthal sample tend to be more retroverted than the human average (Fig. 5A).

Because in fossil hominins the actual orientation of the pelvis relative to the direction of gravity remains unknown, we explored alternatives to the CPP-based measurement of AO. Fig. S9B shows acetabular orientation in the procrustes-aligned (i.e. sample-centered) specimens, thus using the entire pelvis (rather than CPP alone) as a spatial reference. The acetabula of Neanderthals appear to be more retroverted than those of humans, which is consistent with the results for the CPP-aligned pelves (Fig. 5A). Note that, in the mean pelvic shape resulting from procrustes alignment (Fig. S9B), AO aligns approximately with CPP. We further explored how acetabular orientation relative to the direction of gravity changes with pelvic orientation. Fig. S9C indicates that a posterior pelvic tilt (PT) results in a more vertical alignment of anatomically retroverted acetabula such as those of the Neanderthals. Conversely, anterior PT brings anatomically anteverted acetabula more in line with the direction of gravity (38). These considerations are in line with empirical data (39–41).

Evidence from living subjects (42, 43) suggests that pelvic tilt (PT) and sacral slope (SS) reflect how a specific pelvic morphology is optimally oriented in space under different biomechanical loading conditions. In absence of PT and SS data for fossils, pelvic incidence (PI) is typically used as an anatomical proxy, which characterizes the orientation of the surface of the first sacral vertebra (S1) relative to the acetabular centers and the midpoint of the S1 surface (CPP', Fig. S9B). With reference to physical space, PI represents the sum of PT and SS: PI = PT + SS. In humans, large PI angles tend to be associated with more sloping S1 surfaces (44), and with higher degrees of lumbar lordosis (45) to keep the body in equilibrium during stance.

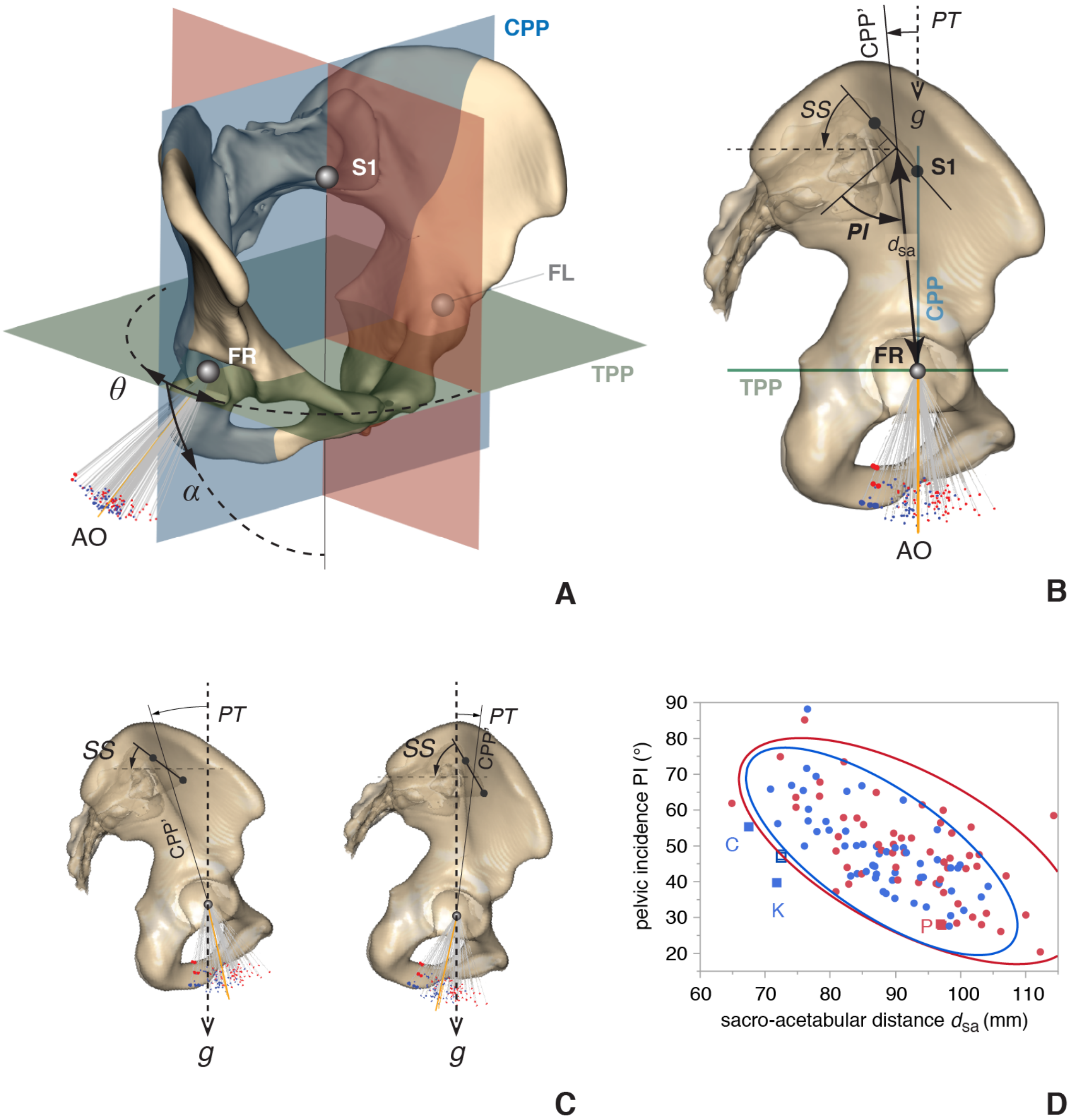

Figure S9. Pelvic reference planes and acetabular orientation. *(A)* The coronal plane (CPP, blue) runs through the right (FR) and left (FL) femoral head centers, and through the anteriormost midpoint of the first sacral vertebra (S1). This plane is approximately vertical during stance and serves as a reference for measuring acetabular ante-/retroversion θ. The transverse plane (TPP) is perpendicular to CPP and serves as a reference for acetabular inclination α. *(B)* Acetabular orientation (AO) in the Procrustes-aligned specimens (red/blue dots: females/males; large/small dots: Neanderthals/humans). Blue and green lines indicate CPP and TPP of graph *A*. CPP is assumed here to be aligned with gravity (*g*). Mean AO (orange line) approximately aligns with CPP (θ=0). Pelvic incidence (PI) is defined as the angle formed between the normal vector of the surface of the first sacral vertebra and the vector pointing from the femoral head center to the midpoint of the S1 surface (plane CPP'). Pelvic tilt (PT) is measured relative to CPP', and sacral slope (SS) relative to the horizontal plane (normal to *g*). (*C*) Changes in pelvic tilt influence acetabular orientation relative to the direction of gravity. A posteriorly tilted pelvis (left graph) tends to align retroverted acetabula (like those of Neanderthals) along the direction of gravity. For any given PI value, posterior tilt requires less lumbar lordosis to maintain the body in equilibrium during stance. (*D*) Pelvic incidence (PI) decreases with increasing sacroacetabular distance (d_{sa}). Compared to humans, Neanderthals have small PI angles for any given d_{sa}. C: La Chapelle-aux-Saints, data from ref. (46); K: Kebara; P: Palomas.

It seems straightforward to use these correlations for inferences about the pelvic orientation and locomotor properties of fossil (45). However, some caution is warranted. In terms of geometry, PI is fully defined geometrically by the orientation of the S1 plane relative to the CPP' plane (Fig. S9B). In terms of pelvic morphology, however, PI correlates with sacro-acetabular distance (d_{sa}; see Fig. S9B and D): short d_{sa} distances imply large PI angles. While our results confirm that the pelvic incidence of the Neanderthals was within the human range of variation (46), they also show that Neanderthals had lower PI angles than humans for any given sacroacetabular distance.

11. Pelvic shape and locomotor biomechanics

The biomechanical properties of the pelvis in conjunction with the proximal femur relevant for stance and bipedal locomotion were explored by computing moment arm proxies for the major muscle groups and for body weight (Table S5, Fig. S10). To this end, 3D surface models of the right proximal femur were generated for all individuals in the adult modern human sample. Also, surface models of the reconstructed Palomas and Tabun Neanderthal femora were generated. The Kebara fossil only preserves a fragmentary left proximal femur. We used the well-preserved right femur of the Neanderthal type specimen as a placeholder, given the similarity of the ilio-acetabular dimensions of the two fossils. Each pelvis in the adult sample was then oriented such that its main axis (defined by the intersection of the coronal plane with the midsagittal plane; see Fig. 5A and Fig. S10A) was aligned vertically, i.e. along the direction of gravity during stance. Additionally, the right femur of each specimen was rotated in its acetabular socket so the shaft axis pointed along the direction of gravity, and the most anterior point on the greater trochanter pointed laterally. Various distances were then measured from femoropelvic anatomical landmarks in mediolateral, anteroposterior and superoinferior directions relative to the femoral head center (FHC), the coronal plane and the midsagittal plane. These measurements serve as proxies for flexor/extensor and abductor moment arm lengths, as specified in Table S5 and Fig. S10A. Furthermore, femoral head radius, neck length and neck-shaft angle were evaluated as additional variables.

The anatomical moment arm length r of the abductors can be estimated by the distance of the femoral head center from the line of action of the abductor muscles (see variable #17 in Fig. S10A). In absence of actual soft tissue data, we used the line from the lateralmost point on the greater trochanter to the ischial tubercle as a first estimate of the line of action. Using this line yields a maximum estimate r_{max}, because the actual line of action intersects the iliac blade at a point inferior to the iliac tubercle. Comparing our estimates (mean values of r_{max} for females and males: 59.2 mm and 66.3 mm) with actual r values from ref. (47) (female and male mean values of r: 52.2 mm and 59.6 mm, respectively) yields r/r_{max} ratios of 0.88 (females) and 0.90 (males). We thus use a scaling factor of 0.89 to compute $r = 0.89\ r_{max}$. Finally, the effective mechanical advantage (EMA) of the abductors is computed as r / R, where R is the distance of the femoral head center from the midsagittal plane (see variable #18 in Fig. S10A).

Neanderthal-human differences in these variables were assessed as follows: all variables were standardized for the effects of sex and pelvic size by regressing them on sex and log centroid size, and keeping the residuals for further analyses. Given the small Neanderthal sample, comparison of means via standard analysis of variance was not possible. Instead, 1000 permuted data sets were generated by shuffling species attributions, and the permuted mean differences between species were compared with the actual mean differences. Analogous analyses were performed for sex differences in these variables (human sample alone), correcting for size effects and shuffling sex attributions. The results of both analyses are summarized in Table S5; significance levels were adjusted by applying a Benjamini-Hochberg false discovery rate (FDR) correction to the original p-values (48). Effect sizes (expressed in z-scores) are generally larger for species differences than for sex differences (Fig. S10B).

Table S5. Femoropelvic measurements and moment arm proxies.

function	var. #	direction	landmarks	reference plane	grand mean	Neanderthal – human contrast			female – male contrast		
						p	z diff.	diff.	p	z diff.	diff.
flexors	1	anterior	ant. sup. iliac spine	CPP	59.2	0.34	-0.35	-2.9	0.22	-0.23	-1.9
	2	anterior	ant. inf. iliac spine	CPP	38.2	0.03	-0.84	-4.3	0	-0.52	-2.8
	3	anterior	pubic symphysis	CPP	47.4	0.68	0.15	1	0	-0.61	-4.3
	4	posterior	lesser trochanter	CPP	21.5	0.76	-0.11	-0.5	0.02	-0.48	-2.2
	5	inferior	lesser trochanter	CPP	59.5	0	-1.57	-7.1	0	-0.95	-4.9
extensors	6	posterior	sacral vertebra 5	CPP	81.7	0	-1.83	-15.1	0.19	0.27	2.2
	7	posterior	ischial tuberosity	CPP	56.0	0	-2.61	-18.5	0.02	-0.41	-3.0
abductors	8	lateral	ant. sup. iliac spine	MPP	114.3	0	1.57	12.7	0.12	-0.3	-2.4
	9	lateral	iliac tubercle	MPP	135.7	0	1.84	10.6	0.02	-0.48	-2.8
	10	lateral	greater trochanter	FHC	59.0	0.01	0.95	6.3	0	-0.59	-4.1
	11	lateral	greater trochanter	MPP	154.3	0	2.2	10.6	0	-1.37	-9.1
	12	superior	iliac tubercle	TPP	106.8	0	-1.86	-13.9	0	-0.86	-7.1
	13	inferior	greater trochanter	TPP	22.7	0	-1.27	-7.4	0.05	-0.41	-2.4
	14		fem. head radius		22.7	0.2	-0.48	-0.6	0	-1.28	-2.2
	15		fem. neck length		53.2	0.46	-0.3	-1.3	0	-1.04	-5.3
	16		fem. neck-shaft angle (°)		132.6°	0	-2.93	-14.9	0.01	0.52	2.7
	17		abductor moment arm length		63.5	0	1.3	4.5	0	-1.43	-7.2
body weight	18	medial	fem. head center	MPP	85.0	0	2.32	7.8	0.89	-0.03	-0.1
EMA	19		var17 / var18		0.66	0.54	-0.23	-0.01	0	-1.18	-0.05

Variables #1-#13 represent linear distances that serve as proxies for moment arm lengths of flexors (rectus femoris, iliopsoas, psoas), extensors (hamstrings and glutaeus maximus), abductors (glutaeus medius and minimus) and body weight. Lengths are measured from landmarks to reference planes along the indicated direction. Neanderthal-human and female-male contrasts are evaluated with the methods described in section S11. Contrasts are characterized by significance level p (green: $p<0.02$), and group-mean difference (z-score difference and absolute difference). Grand means and absolute differences are in mm, except for femoral neck-shaft angle (°) and effective mechanical advantage (EMA, dimensionless).

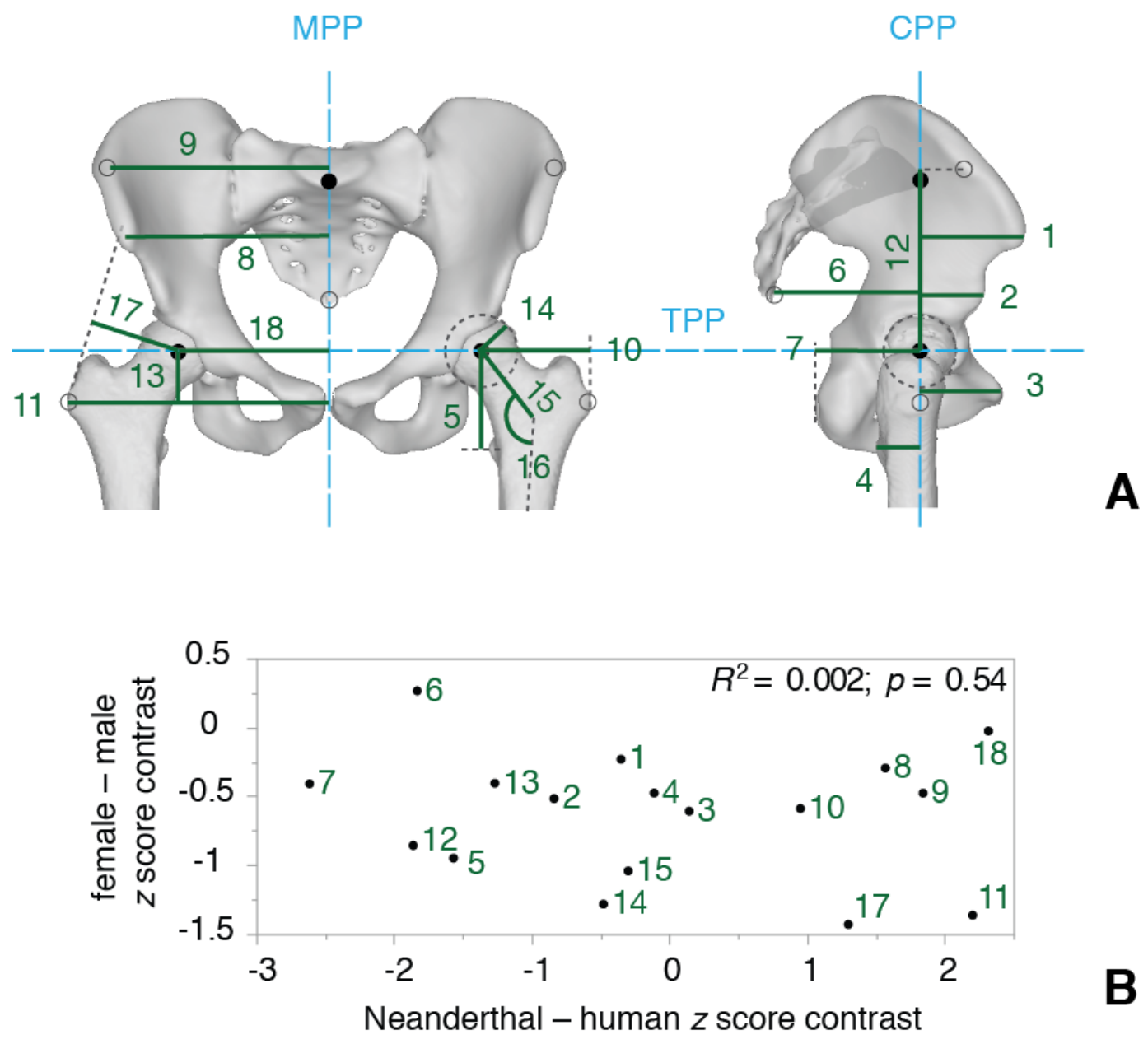

Figure S10. Femoropelvic biomechanical measurements. (*A*) Measurement definitions. Variable numbers (green) refer to the variables listed in Table S5. Measurements are taken from the midsagittal (MPP), coronal (CPP) and transverse (TPP) pelvic planes. CPP runs through the femoral head centers and the anteriormost point of the first sacral vertebra. TPP is perpendicular to CPP and MPP, and runs through the femoral head centers. (*B*) Independence of female-male and Neanderthal-human difference patterns in the linear dimensions shown in (*A*) (*z*-score values see Table S5). Note large range of Neanderthal-human z-score differences compared to female-male differences.

12. Pelvic shape and birth mechanisms

Correlating pelvic shape with birth mechanisms and outcomes has a long tradition, especially the notion that certain "pelvic types" ("android") bear greater risk of birth complications than others ("gynaecoid") (49, 50). The concepts developed by Thoms (49) and Caldwell & Moloy (50) have been highly influential in evolutionary studies of birth (51), but have recently been challenged on various grounds (52, 53). Notably, intrapopulation pelvic shape variation is a continuum that cannot be clearly subdivided into distinct types, and variation between populations is substantial (52, 53), which makes it difficult to ascertain links between shape and obstetric function. Interestingly, similar criticism was already expressed during the discussion that followed Caldwell and Moloy's 1938 presentation at the Royal Society of Medicine; see ref. (50), p. 30:

> "Professor PRESTON MAXWELL said that in the cases with which he and his colleagues had to deal in Peiping, it was a matter of considerable difficulty to decide into what class pelves should be placed. […] Dr. CLARK NICHOLSON said that his criticisms of Dr. Moloy's paper were based on radiological measurements on more than 350 cases in a cottage hospital. He thought that Dr. Moloy had made too little mention of racial differences in the pelvis. … He (the speaker) thought that a classification of the pelvis which depended solely on appearances, and not on accurate measurements, was not truly scientific and could not be of permanent value in obstetrics. His figures tended to show that there was no close relationship between the shape of the pelvis and difficulty in labour."

Since the recent critical reevaluations (52, 53) did not examine the original data statistically, we present a reanalysis of the datasets published in (49) and (50). Using standard Chi-square statistics, the Thoms data (49) show no significant effect of pelvic type on birth outcomes (see Table S6A). The Caldwell and Moloy raw data (50) (Table S6B) cannot be directly submitted to standard statistical tests, because of the peculiar sampling scheme. These authors chose sample sizes of N=100 for each category of birth outcome without mentioning how sampling was performed, and without reporting the associated population frequencies of pelvic types in the total sample. Therefore, any direct statistical evaluation of the effect of pelvic type on birth outcomes is based on inflated total numbers of problematic versus spontaneous births. Nevertheless, it is possible to estimate the expected proportion of interventional (B_i) to spontaneous births (B_s). A large sample of spontaneous-onset births (54) exhibits a B_s/B_i ratio of 3.2. Similarly, the much smaller Thoms sample exhibits a B_s/B_i ratio of 3.4. Adjusting the marginal totals of the Caldwell and Moloy data to this ratio results in Table S6C, which shows no significant effect of pelvic type on birth outcome.

Table S6. Statistical reevaluation of pelvic typology and birth outcomes

Table S6 A. Thoms 1937: original data from ref. (49)

pelvic type	*spontaneous*	*operative*	*breech*	*occ-post*	*sum interventional*
dolichopellic	39	11	7	13	31
mesatipellic	138	37	6	22	65
brachypellic	105	31	7	13	51
platypellic	12	7	0	2	9

Chi-square on original table: 13.92; DF = 9; $p = 0.125$
Chi-square on spontaneous vs. interventional births: 4.38; DF = 3; $p = 0.223$

Table S6 B. Caldwell and Moloy 1938: original data from ref. (50)

pelvic type	*spontaneous*	*low forceps*	*low mid forceps*	*mid forceps*	*C section*	*total interventional (forceps and C-sec)*	B_s/B_i
anthropoid	25	31	23	14	16	84	1.30
android	27	31	47	56	57	191	1.14
gynaecoid	37	32	12	15	12	71	1.52
platypelloid	11	6	18	15	15	54	1.20
sum	100	100	100	100	100	400	1.25

Table S6 C. Caldwell and Moloy 1938 data, adjusted to realistic marginal totals $B_s/B_i = 3.2$

pelvic type	*original data: spontaneous (B_s)*	*original data: interventional (B_i)*	*adjusted data: interventional*
anthropoid	25	84	7
android	27	191	15
gynaecoid	37	71	6
platypelloid	11	54	4
sum	100	400	32

Chi-square: 5.63; DF = 3; $p = 0.131$

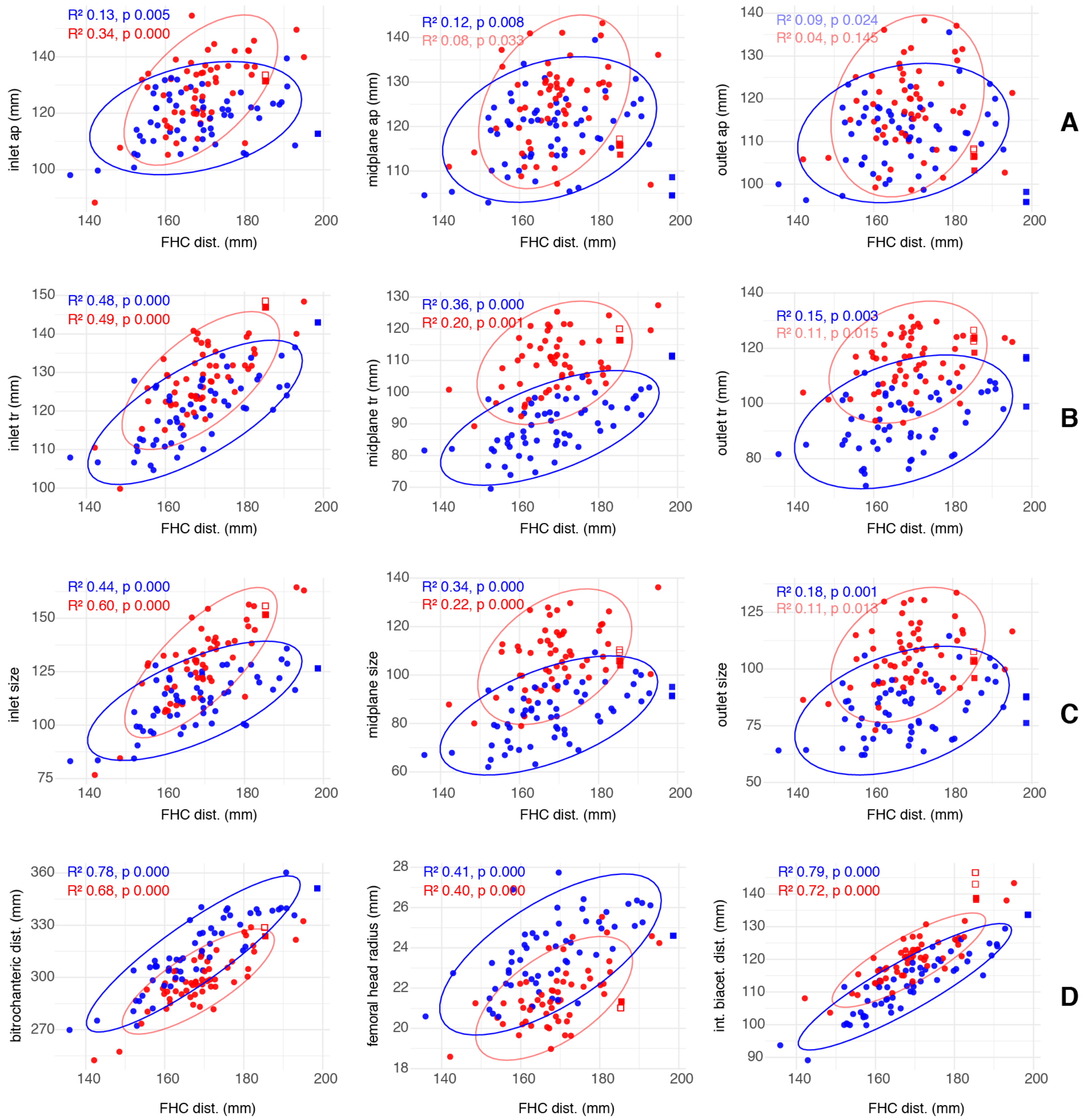

Figure S11. Pelvic dimensions and their relationship with the distance between femoral head centers (FHC distance). Red/blue symbols: females/males; circles: *H. sapiens*; squares *H. neanderthalensis*; filled: Palomas, Kebara; open: Tabun, Kebara reconstruction from ref. (20). 95% ellipsoids and associated R^2 and p values are shown for human females and males. **(*A-C*) Obstetric dimensions.** (*A*) Anteroposterior inlet, midplane and outlet dimensions (ap); (*B*) transverse dimensions (tr); (*C*) area sizes. In both sexes, linear and area dimensions increase with increasing FHC distance, but sex-specific distributions are clearly distinct, with females exhibiting larger obstetric dimensions relative to FHC distance than males. **(*D*) Femoropelvic dimensions.** Left graph: bitrochanteric distance (measured between lateralmost points on left and right greater trochanters); middle graph: femoral head radius; right graph: internal biacetabular distance (measured between medialmost points on left and right acetabular surfaces). Females have relatively smaller bitrochanteric and femoral head dimensions than males. However, females have relatively larger internal biacetabular distance. This is partly due to their smaller femoral head (and acetabular) dimensions, which permit larger transverse midplane and outlet dimensions (*B*).

13. Obstetric and locomotor constraints

A notable finding from the analyses presented in Fig. 5 and Table S5 is that the mean lateral distance of the femoral head center (FHC) from the midsagittal plane is similar in both sexes. Given the biomechanical importance of FHC distance – the anatomical moment arm of body weight during stance – we performed detailed analyses of FHC distance and its relation with obstetrically and biomechanically relevant dimensions of the pelvis (see Fig. S11).

14. Pelvic size, shape and the thermoregulation hypothesis

We expand Ruff's standard model of cylindrical body form (55) to a model that accounts for different anteroposterior and mediolateral dimensions, representing the major and minor axes of an ellipse that forms the basis of a cylindroid (Fig. S12A). We computed the minimum ellipse that comprises the lateralmost, anteriormost and posteriormost points of the femoropelvic morphology projected onto the body's transverse plane (Fig. S12A). The height h of the cylindroid is given by the vertical distance from the lesser trochanter to the highest point on the iliac crest. The surface of the cylindroid is the product of ellipse circumference C and height h, where C is evaluated with the Ramanujan approximation:

$$S = C\,h = \pi\,[3(a+b) - ((3a+b)(a+3b))^{-1/2}]\,h,$$

where a and b are the ellipses major radii. The volume of the cylindroid is the product of ellipse area A and height h:

$$V = A\,h = \pi\,a\,b\,h.$$

Note that the surface-to-volume ratio of the cylindroid is equivalent to the circumference-to-area ratio:

$$S/V = (C\,h) / (A\,h) = C/A$$

Fig. S12B shows a double-logarithmic plot of S/V versus V, where V is a proxy of pelvic size. Humans exhibit sex-specific distributions, with females having slightly smaller S/V ratios in relation to their pelvic size than males. In both sexes, the double-logarithmic regression slopes are close to the expected value for an Euclidean body ($S/V \sim V^{-1/3}$). Neanderthals fall well within the respective human female and male distributions, thus providing no support for the heat-conservation hypothesis based on S/V minimization.

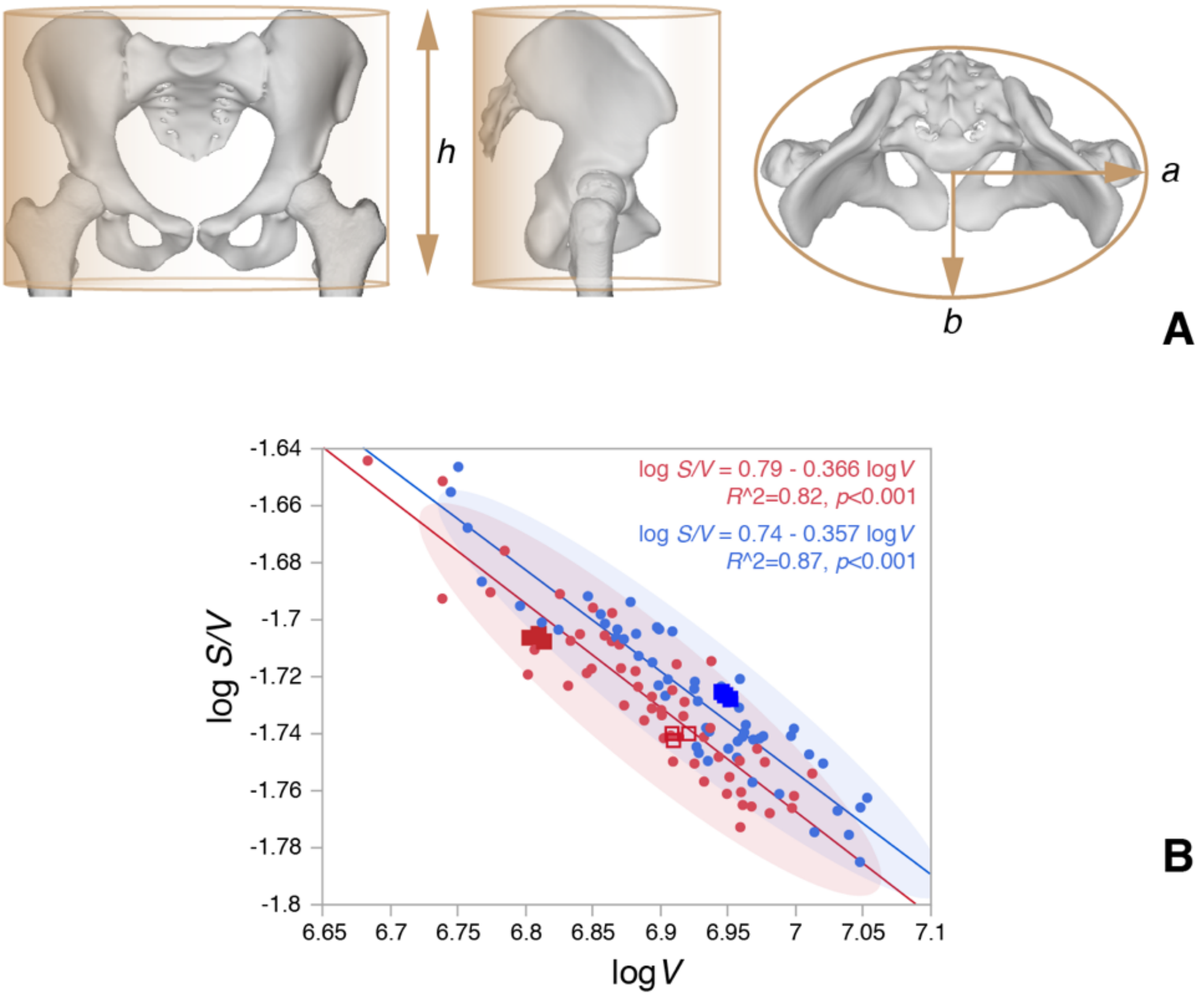

Figure S12. Pelvic surface-to-volume ratios in humans and Neanderthals. (*A*) Volume *V* and surface area *S* of the pelvic girdle region are approximated by a cylindroid hull with major axis radii *a* and *b*, and height *h*. (*B*) Double-logarithmic plot of surface-to-volume ratio (*S*/*V*) versus volume (*V*). Circles/squares: humans/Neanderthals; red/blue: females/males; 95% density ellipses and regression equations for human sex-specific distributions. Details see text S14.

Dataset S1 Caption.

3D landmark coordinates, centroid sizes and metadata of adult pelves. Data are size-normalized, symmetrized and Procrustes-aligned. Landmark descriptions see Table S2A.

Dataset S2 Caption.

3D landmark coordinates, centroid sizes and metadata of immature pelves. Data are size-normalized, symmetrized and Procrustes-aligned. Landmark descriptions see Table S2B.

SI References

1. T. Akazawa, S. Muhesen, Y. Dodo, O. Kondo, Y. Mizoguchi, Neanderthal infant burial. *Nature* **377**, 585–586 (1995).
2. M. S. Ponce de León, *et al.*, Neanderthal brain size at birth provides insights into the evolution of human life history. *Proc Natl Acad Sci USA* **105**, 13764–13768 (2008).
3. M. J. Walker, J. Ortega, K. Parmová, M. V López, E. Trinkaus, Morphology, body proportions, and postcranial hypertrophy of a female Neandertal from the Sima de las Palomas, southeastern Spain. *Proc Natl Acad Sci U S A* **108**, 10087–10091 (2011).
4. E. Trinkaus, "The Palomas Neanderthal sample" in *The People of Palomas, Neandertals from the Sima de Las Palomas Del Cabezo Gordo, Southeastern Spain*, E. Trinkaus, M. J. Walker, Eds. (Texas A&M University Press, 2017), pp. 19–23.
5. E. Trinkaus, K. A. Robson, J. Ortega, K. Karaková, "The Palomas postcrania" in *The People of Palomas, Neandertals from the Sima de Las Palomas Del Cabezo Gordo, Southeastern Spain*, E. Trinkaus, M. J. Walker, Eds. (Texas A&M University Press, 2017), pp. 183–228.
6. M. J. Walker, *et al.*, Late Neandertals in Southeastern Iberia: Sima de las Palomas del Cabezo Gordo, Murcia, Spain. *Proc Natl Acad Sci USA* **105**, 20631–20636 (2008).
7. S. O. Rasmussen, *et al.*, A stratigraphic framework for abrupt climatic changes during the Last Glacial period based on three synchronized Greenland ice-core records: refining and extending the INTIMATE event stratigraphy. *Quat. Sci. Rev.* **106**, 14–28 (2014).
8. J. S. Carrión, *et al.*, Glacial refugia of temperate, Mediterranean and Ibero-North African flora in south-eastern Spain: New evidence from cave pollen at two Neanderthal man sites. *Glob. Ecol. Biogeogr.* **12**, 119–129 (2003).
9. J. S. Carrión García, *et al.*, "Fitodiversidad arbórea en el litoral del sureste español durante el Pleistoceno Superior" in *Geomorfologia Litoral i Quaternari - Homenatge Al Profesor Vicenç Rosselló i Verger*, E. Sanjaume, J. F. Mateu, Eds. (Universitat de València, 2005), pp. 103–112.
10. M. J. Walker, *et al.*, Preliminary dating of deep layers at Sima de las Palomas del Cabezo Gordo (Torre Pacheco, Murcia, Spain) in *Proceedings of the European Society for the Study of Human Evolution*, (2017), p. 210.
11. J. A. Sánchez-Cabeza, J. García-Orellana, L. Gibert, "Uranium-thorium dating of natural carbonates: application to the Cabezo-Gordo site (Murcia, Spain)." in *The Hominids and Their Environment during the Lower and Middle Pleistocene of Eurasia*, J. Gibert, Sánchez F., L. Gibert, F. Ribot, Eds. (Museo de Prehistoria y Paleontología "J. Gibert," 1999), pp. 261–268.
12. M. J. Walker, "Nuevas perspectivas paleoantropológicas en la Región de Murcia: La Cueva Negra del Estrecho del Río Quípar en Caravaca de la Cruz y la Sima de las Palomas del Cabezo Gordo en Torre Pacheco" in *Paleolítico: De Los Primeros Pobladores Al Ocaso Neandertal En La Península Ibérica*, M. A. Bernal Gómez, A. Santiago Pérez, Eds. (Editorial Almuzara, 2021), pp. 231–253.
13. A. Fedorov, *et al.*, 3D Slicer as an image computing platform for the quantitative imaging network. *Magn Reson Imaging* **30**, 1323–1341 (2012).
14. D. A. E. Garrod, D. M. A. Bate, T. D. McCown, A. Keith, Royal College of Surgeons of England., *The stone age of Mount Carmel* (The Clarendon Press, 1937).
15. Y. Rak, B. Arensburg, Kebara 2 Neanderthal pelvis: first look at a complete inlet. *Am J Phys Anthr.* **73**, 227–231 (1987).
16. T. D. Weaver, J. J. Hublin, Neandertal birth canal shape and the evolution of human childbirth. *Proc Natl Acad Sci USA* **106**, 8151–8156 (2009).
17. C. P. E. Zollikofer, M. S. Ponce de León, Reconsidering Neanderthal birth: Tabun mom, Kebara dad, Mezmaiskaya baby, and the consequences. *Amercian J. Phys. Anthropol.* **141**, 252 (2010).
18. M. W. Grabowski, J. D. Polk, C. C. Roseman, Divergent patterns of integration and reduced constraint in the human hip and the origins of bipedalism. *Evolution (N. Y).* **65**, 1336–1356

(2011).
19. A. Huseynov, *et al.*, Developmental evidence for obstetric adaptation of the human female pelvis. *Proc Natl Acad Sci U S A* **113**, 5227–5232 (2016).
20. M. T. Adegboyega, P. A. Stamos, J. J. Hublin, T. D. Weaver, Virtual reconstruction of the Kebara 2 Neanderthal pelvis. *J. Hum. Evol.* **151**, 102922 (2021).
21. I. L. Dryden, K. Mardia, *Statistical Shape Analysis* (John Wiley & Sons, 1998).
22. D. C. Adams, E. Otarola-Castillo, geomorph: an R package for the collection and analysis of geometric morphometric shape data. *Methods Ecol Evol* **4**, 393–399 (2013).
23. S. Schlager, Morpho: Calculations and visualisations related to Geometric Morphometrics. http://sourceforge.net/projects/morpho-rpackage. (2014).
24. J. L. Arsuaga, *et al.*, A complete human pelvis from the Middle Pleistocene of Spain. *Nature* **399**, 255–258 (1999).
25. S. W. Simpson, *et al.*, A female *Homo erectus* pelvis from Gona, Ethiopia. *Science* **322**, 1089–1092 (2008).
26. J. M. Kibii, *et al.*, A partial pelvis of *Australopithecus sediba*. *Science* **333**, 1407–1411 (2011).
27. R. Crompton, *et al.*, Bipedalism or bipedalisms: The os coxae of StW 573. *J. Anat.* **248**, 163–183 (2026).
28. E. Stansfield, B. Fischer, N. D. S. Grunstra, M. V. Pouca, P. Mitteroecker, The evolution of pelvic canal shape and rotational birth in humans. *BMC Biol.* **19**, 1–11 (2021).
29. A. G. Warrener, Hominin hip biomechanics: changing perspectives. *Anat. Rec.* **300**, 932–945 (2017).
30. J. Y. Lazennec, A. Brusson, M. A. Rousseau, Lumbar-pelvic-femoral balance on sitting and standing lateral radiographs. *Orthop. Traumatol. Surg. Res.* **99**, S87–S103 (2013).
31. C. Boulay, *et al.*, Anatomical reliability of two fundamental radiological and clinical pelvic parameters: incidence and thickness. *Eur. J. Orthop. Surg. Traumatol.* **15**, 197–204 (2005).
32. C. Tardieu, K. Hasegawa, M. Haeusler, How did the pelvis and vertebral column become a functional unit during the transition from occasional to permanent bipedalism? *Anat. Rec.* **300**, 912–931 (2017).
33. A. B. Lawrence, A. S. Hammond, C. V. Ward, Acetabular orientation, pelvic shape, and the evolution of hominin bipedality. *J. Hum. Evol.* **200**, 103633 (2025).
34. S. W. Higgins, *et al.*, A novel approach for determining three-dimensional acetabular orientation: Results from two hundred subjects. *J. Bone Jt. Surg. - Am. Vol.* **96**, 1776–1784 (2014).
35. H. Zhang, *et al.*, Three-dimensional acetabular orientation measurement in a reliable coordinate system among one hundred Chinese. *PLoS One* **12**, e0172297 (2017).
36. E. P. Tannenbaum, *et al.*, A computed tomography study of gender differences in acetabular version and morphology: Implications for femoroacetabular impingement. *Arthrosc. - J. Arthrosc. Relat. Surg.* **31**, 1247–1254 (2015).
37. D. Suzuki, S. Nagoya, H. Takashima, K. Tateda, T. Yamashita, Three-dimensional orientation of the acetabulum. *Clin. Anat.* **30**, 753–760 (2017).
38. R. K. Zahn, *et al.*, Pelvic tilt compensates for increased acetabular anteversion. *Int. Orthop.* **40**, 1571–1575 (2016).
39. S. K. Song, *et al.*, Changes of acetabular anteversion according to pelvic tilt on sagittal plane under various acetabular inclinations. *J. Orthop. Res.* **39**, 806–812 (2021).
40. J. Schwarz, *et al.*, Effect of pelvic sagittal tilt and axial rotation on functional acetabular orientation. *Orthopedics* **46**, E27–E30 (2023).
41. K. Vasiljeva, *et al.*, Dynamic acetabular cup orientation during gait: A study of fast- and slow-walking total hip replacement patients. *Bioengineering* **11**, 151 (2024).
42. J. W. Day, G. L. Smidt, T. Lehmann, Effect of pelvic tilt on standing posture. *Phys. Ther.* **64**, 510–516 (1984).
43. P. G. Kroll, S. Arnofsky, S. Leeds, D. Peckham, A. Rabinowitz, The relationship between lumbar lordosis and pelvic tilt angle. *J. Back Musculoskelet. Rehabil.* **14**, 21–25 (2000).
44. G. Vaz, P. Roussouly, E. Berthonnaud, J. Dimnet, Sagittal morphology and equilibrium of pelvis and spine. *Eur. Spine J.* **11**, 80–87 (2002).
45. C. Boulay, *et al.*, Sagittal alignment of spine and pelvis regulated by pelvic incidence:

Standard values and prediction of lordosis. *Eur. Spine J.* **15**, 415–422 (2006).
46. M. Haeusler, *et al.*, Morphology, pathology, and the vertebral posture of the La Chapelle-aux-Saints Neandertal. *Proc. Natl. Acad. Sci. U. S. A.* **116**, 4923–4927 (2019).
47. A. G. Warrener, K. L. Lewton, H. Pontzer, D. E. Lieberman, A wider pelvis does not increase locomotor cost in humans, with implications for the evolution of childbirth. *PLoS One* **10**, e0118903 (2015).
48. Y. Benjamini, Y. Hochberg, Controlling the false discovery rate: A practical and powerful approach to multiple testing. *J. R. Stat. Soc. Ser. B* **57**, 289–300 (1995).
49. H. Thoms, Obstetrical significance of pelvic variations. *Br. Med. J.* **2**, 210 (1937).
50. W. E. Caldwell, H. C. Moloy, Anatomical variations in the female pelvis: their classification and obstetrical significance. *Proc. R. Soc. Med.* **32**, 1–30 (1938).
51. K. Rosenberg, W. Trevathan, Birth, obstetrics and human evolution. *Int J Obs. Gynaecol* **109**, 1199–1206 (2002).
52. L. Betti, Shaping birth: variation in the birth canal and the importance of inclusive obstetric care. *Philos. Trans. R. Soc. B Biol. Sci.* **376**, 20200024 (2021).
53. C. VanSickle, K. L. Liese, J. N. Rutherford, Textbook typologies: Challenging the myth of the perfect obstetric pelvis. *Anat. Rec.* **305**, 952–967 (2022).
54. P. Martin, *et al.*, Timing of singleton births by onset of labour and mode of birth in NHS maternity units in England, 2005–2014: A study of linked birth registration, birth notification, and hospital episode data. *PLoS One* **13**, e0198183 (2018).
55. C. B. Ruff, Climate and body shape in hominid evolution. *J Hum Evol* **21**, 81–104 (1991).
